# Identification and Characterization of Flavonoid Biosynthetic Enzyme Genes in *Salvia miltiorrhiza* (Lamiaceae)

**DOI:** 10.3390/molecules23061467

**Published:** 2018-06-16

**Authors:** Yuxing Deng, Caili Li, Heqin Li, Shanfa Lu

**Affiliations:** 1 Institute of Medicinal Plant Development, Chinese Academy of Medical Sciences & Peking Union Medical College, No.151 Malianwa North Road, Haidian District, Beijing 100193, China; yuxingdeng2016@163.com (Y.D.); licaili390@163.com (C.L.); hqliaau@163.com (H.L.); 2 College of Agronomy, Qingdao Agricultural University, No. 700 Changcheng Road, Chengyang District, Qingdao 266109, China

**Keywords:** flavonoid, methyl jasmonate, *Salvia miltiorrhiza*, traditional Chinese medicine

## Abstract

Flavonoids are a class of important secondary metabolites with a broad spectrum of pharmacological functions. *Salvia miltiorrhiza* Bunge (Danshen) is a well-known traditional Chinese medicinal herb with a broad diversity of flavonoids. However, flavonoid biosynthetic enzyme genes have not been systematically and comprehensively analyzed in *S. miltiorrhiza*. Through genome-wide prediction and molecular cloning, twenty six flavonoid biosynthesis-related gene candidates were identified, of which twenty are novel. They belong to nine families potentially encoding chalcone synthase (CHS), chalcone isomerase (CHI), flavone synthase (FNS), flavanone 3-hydroxylase (F3H), flavonoid 3′-hydroxylase (F3′H), flavonoid 3′,5′-hydroxylase (F3′5′H), flavonol synthase (FLS), dihydroflavonol 4-reductase (DFR), and anthocyanidin synthase (ANS), respectively. Analysis of intron/exon structures, features of deduced proteins and phylogenetic relationships revealed the conservation and divergence of *S. miltiorrhiza* flavonoid biosynthesis-related proteins and their homologs from other plant species. These genes showed tissue-specific expression patterns and differentially responded to MeJA treatment. Through comprehensive and systematic analysis, fourteen genes most likely to encode flavonoid biosynthetic enzymes were identified. The results provide valuable information for understanding the biosynthetic pathway of flavonoids in medicinal plants.

## 1. Introduction

Flavonoids, a class of important secondary metabolites, are widely distributed in the plant kingdom. Flavonoids contain a fifteen-carbon atom backbone consisting of two phenyl rings (A and B) and a heterocyclic pyran ring (C). The C_15_ backbone is abbreviated as C_6_–C_3_–C_6_. Based on the oxidation and saturation status of the C ring, flavonoids are classified into different subgroups, mainly including flavones, flavonols, flavanones, flavanols, isoflavones, aurones, anthocyanins, and proanthocyanidins (PA, also called condensed tannins) [1,2]. Flavonoids play a variety of physiological roles in plant growth, development, and reproduction. They act as the most important pigment in flower petals to attract pollinators and are involved in UV protection (UV-B) and symbiotic nitrogen fixation. They also play significant roles in plant defense against phytopathogens and in auxin transport regulation [1,3]. In addition, flavonoids are important bioactive compounds with nutritional and medicinal benefits for humans due to their diverse biological and pharmacological activities in hepato protection, anti-oxidation, anti-mutagenesis, anti-cancer, anti-inflammation, anti-bacterial, anti-viral, and against coronary heart diseases [1].

**Figure 1:**
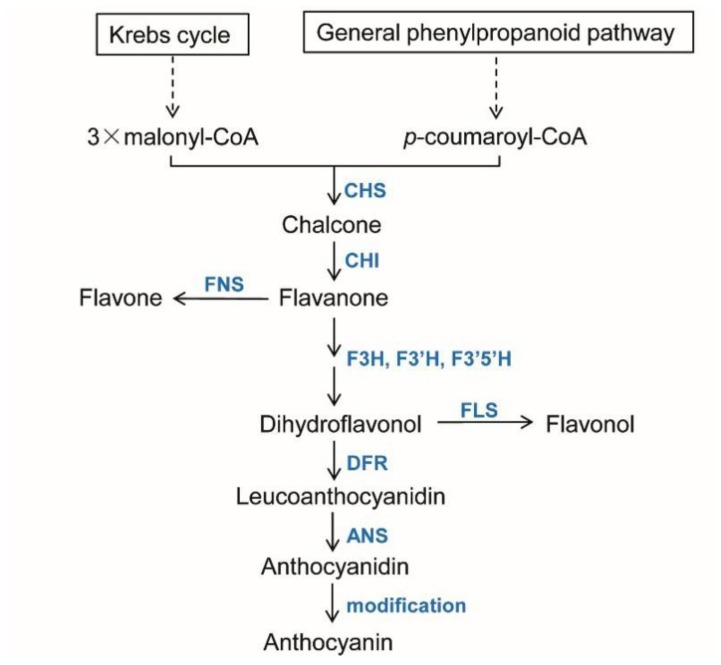
A schematic view of the biosynthetic pathways of flavonoids. The biosynthesis of flavonoids begins with the condensation of one molecule of p-coumaroyl-CoA derived from the general phenylpropanoid pathway (GPP) and three molecules of malonyl-CoA from the krebs cycle. Key enzymes are shown in blue letters. The Krebs cycle and the general phenylpropanoid pathway (GPP) are indicated in boxes with solid black lines. Dashed arrows denote multiple steps. Solid arrows represent single biosynthetic steps. CHS, chalcone synthase; CHI, chalcone isomerase; F3H, flavanone 3-hydroxylase; F3′H, flavonoid 3′-hydroxylase; F3′5′H, flavonoid 3′,5′-hydroxylase; DFR, dihydroflavonol reductase; ANS, anthocyanidin synthase; FLS, flavonol synthase; FNS, flavone synthase.

The biosynthetic pathway of flavonoids has been generally elucidated from studies in numerous plant species (Figure 1). Thus, enzymes catalyzing flavonoid biosynthesis have been analyzed in various plant species, such as *Arabidopsis thaliana* [4], *Glycine max* [5], and *Vitis vinifera* [3]. *Chalcone synthase* (CHS, EC 2.3.1.74) acts in the first step of the flavonoid biosynthetic pathway. It catalyzes the iterative condensation and subsequent intramolecular cyclization of one p-coumaroyl-CoA with three acetate residues from malonyl-CoA molecules to form chalcone [6]. In the second step, chalcone isomerase (CHI, EC 5.5.1.6) catalyzes the stereospecific isomerization of chalcone into flavanone [7]. Thereafter, flavone synthase (FNS, EC 1.14.11.22) introduces a double bond between the C2 and C3 positions of flavanone, converting flavanone into flavone. It is noteworthy that there are two types of plant FNS, including FNSI and FNSII [8]. FNSI mainly exists in Apiaceae plants, such as parsley [9]. FNSII is much more widespread. It has been found in various plant families, such as Lamiaceae, Asteraceae, Plantaginaceae, and Leguminosae [8]. Flavanone 3-hydroxylase (F3H, EC 1.14.11.9), also termed flavanone 3β-hydroxylase (FHT), catalyzes the 3-hydroxylation of flavanone to form dihydroflavonol [9]. Flavonoid 3′-hydroxylase (F3′H, EC 1.14.13.21) and flavonoid 3′, 5′-hydroxylase (F3′5′H, EC 1.14.13.88) catalyze the hydroxylation of the B ring of flavonoids at the 3′ and the 3′ 5′-position, respectively [10]. Flavonol synthase (FLS, EC 1.14.11.23) catalyzes the desaturation of dihydroflavonol into flavonol [9]. Competing with FLS for the same substrate, dihydroflavonol 4-reductase (DFR, EC 1.1.1.219) catalyzes stereospectic reduction of dihydroflavonol into leucoanthocyanidin [11]. Anthocyanidin synthase (ANS, EC 1.14.11.19), also termed leucoanthocyanidin dioxygenase (LDOX), catalyzes the conversion of leucoanthocyanidin into anthocyanidin [9].

*Salvia miltiorrhiza*, also known as Danshen in Chinese, is a perennial herb from the Lamiacae. It is one of the most popularly used traditional Chinese medicines (TCMs), with notable effects in treating cardiovascular diseases [12]. In addition, *S. miltiorrhiza* is a model medicinal plant species with the whole genome sequence and genetic transformation system available [12,13,14,15,16]. It has been shown that, in addition to the bioactive compounds, such as phenolic acids and tanshinones, *S. miltiorrhiza* medicinal preparations also contain high content of flavonoids [17]. However, *S. miltiorrhiza* flavonoid biosynthetic enzyme genes have not been systematically studied. Here, genome-wide identification and characterization of flavonoid biosynthetic enzyme genes in *S. miltiorrhiza* are reported.

## 2. Results and Discussion

### 2.1. Prediction and Molecular Cloning of Flavonoid Biosynthesis-Related Genes in S. miltiorrhiza

Using a systematic computational approach, a total of twenty six putative flavonoid biosynthetic enzyme gene models, including twenty that have not been reported before, were predicted from the current genome assembly of *S. miltiorrhiza* (line 99–3) (Table 1). They are members of nine gene families, including *CHS*, *CHI*, *FNS*, *F3H*, *F3′H*, *F3′5′H*, *FLS*, *DFR* and *ANS*. For ANS, FNSII, F3′5′H and DFR, they are encoded by a single gene, whereas F3H and FLS are encoded by two, and CHI, F3′H and CHS are encoded by four, six, and eight genes, respectively. Among the twenty six gene models, twenty four are full-length, whereas the other two are partial (Supplementary Figure S1). 

In order to validate the prediction and obtain full-length sequences of the partial gene models, molecular cloning was carried out using PCR. Full-length open reading frames (ORFs) of the twenty six genes were cloned and sequenced. It verifies all of the predicted gene models. The genes identified were designated as *SmCHS1*–*SmCHS8*, *SmCHI1*–*SmCHI4*, *SmFNSII*, *SmF3H1*, *SmF3H2*, *SmF3′H1*–*SmF3′H6*, *SmF3′5′H*, *SmFLS1*, *SmFLS2*, *SmDFR*, and *SmANS*, respectively. 

BLAST analysis of the cloned cDNAs against the nucleotide collection (nr/nt) database (http://blast.ncbi.nlm.nih.gov/Blast.cgi) using the BLASTn algorithm with default parameters [18] showed that the coding regions of *SmF3′H1*, *SmF3′H2*, *SmF3′H4*, *SmF3′H5*, *SmF3′5′H* and *SmFNSII* shared extremely high similarity (90% identities) with previously reported *S. miltiorrhiza* cytochrome P450 cDNAs assembled from high-throughput RNA-seq data [19]. The other twenty identified genes have not been previously characterized.

### 2.2. SmCHS1–SmCHS8

The *CHSs* are members of the polyketide synthase (PKS) gene superfamily. *CHSs* are ubiquitous in the plant kingdom, having been described from the lower bryophytes to the gymnosperms and angiosperms. For example, *Antirrhinum majus* [20] and *Petroselinum crispum* [21] have one *CHS* gene, whereas *Ipomoea purpurea* [22], *Gerbera hybrida* [23] and *Malus domestica* [24] contain multiple genes with different spatial and temporal expression. In this study, we identified eight *S. miltiorrhiza SmCHS genes*. The deduced amino acid sequences have high sequence identities with CHS or CHS-like proteins from other plant species and contain the conserved chalcone and stilbene synthases domains, including Chal_sti_synt_N (pfam00195) and Chal_sti_synt_C (pfam02797) (Supplementary Figure S2). This is further evidence that the identified *SmCHSs* indeed encode CHS or CHS-like proteins. The ORF length, amino acid number, predicted molecular weight, and theoretical isoelectric point (pI) are shown in Table 1. Gene schematic structure analysis showed that *SmCHS4* had two introns and the other seven *SmCHSs* contained a single intron (Supplementary Figure S1). The results are consistent with those from other plant *CHS* genes [25]. Amino acid sequence comparison of *A. thaliana* AtCHS and SmCHSs to *Medicago sativa* MsCHS2 that has crystal structure available [6], AtCHS and SmCHSs showed that all CHSs contained the catalytic triad Cys164-His303-Asn336 (hereafter residue numbers refer to MsCHS2) and the gatekeeper Phe215 (Supplementary Figure S3). The G372FGPG residue, a CHS signature sequence that provides stereo-control during the cyclization [26], exists in MsCHS2, AtCHS and six SmCHSs including SmCHS1, SmCHS3–SmCHS5, SmCHS7, and SmCHS8. In addition, MsCHS2, AtCHS and SmCHS1 contain Thr197, Gly256 and Ser338, three residues shaping the 4-coumaroly-CoA binding pocket and the polyketide cyclization pocket (Supplementary Figure S3). Those functional residues were replaced by different amino acids in SmCHS2–SmCHS8, indicating divergent enzymatic activities of SmCHSs. 

**Figure 2:**
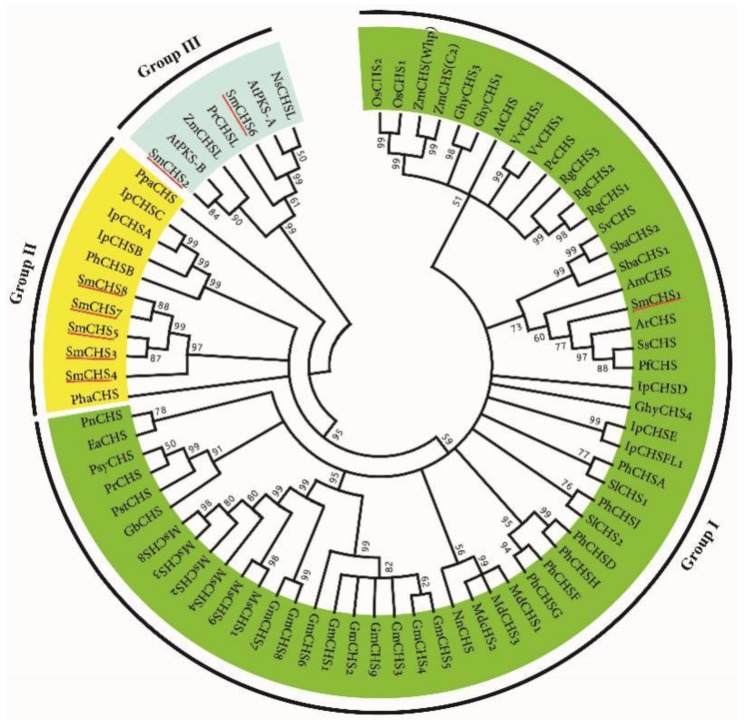
The phylogenetic relationship of CHS and CHS-like proteins. The numbers at the nodes represent the bootstrap values. These CHS protein sequences used for phylogenetic analysis were retrieved from NCBI and their accession numbers are listed in Supplementary Table S4 online.

In order to elucidate the phylogenetic relationship among SmCHSs and CHSs from other plant species, a phylogenetic tree was constructed for 76 CHSs from 30 plant species (Figure 2). Plant CHSs cluster into three groups. Group I is the largest group, containing MsCHS2, AtCHS, VvCHS and various other characterized common CHSs. SmCHS1 is included in group I, indicating it is similar to other more common CHSs. The result is consistent with conserved amino acid residue analysis (Supplementary Figure S3). SmCHS3–SmCHS5, SmCHS7 and SmCHS8 cluster in Group II. This group also include one of the oldest CHSs, *Physcomitrella patens* PpaCHS [27], and three differentially expressed *I. purpurea* IpCHS, IpCHSA, IpCHSB and IpCHSC [22]. SmCHS2 and SmCHS6 are members of group III, a group with anther-specific CHS-like (ASCL) enzymes [28]. It suggests that SmCHS2 and SmCHS6 are probably ASCL proteins. 

**Figure 3:**
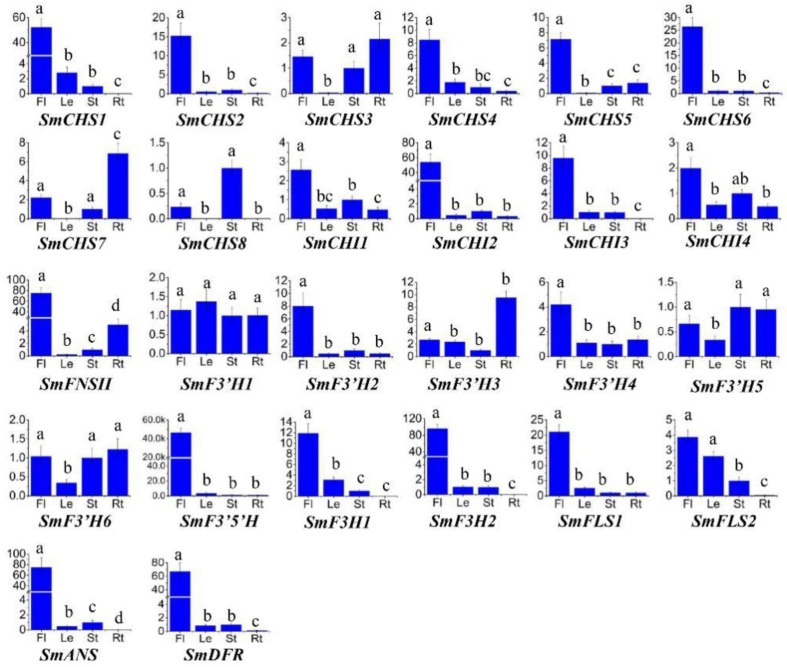
Tissue-specific expression of flavonoid biosynthesis-related genes. The levels of transcripts in flowers (Fl), leaves (Le), stems (St) and roots (Rt) of *S. miltiorrhiza* were analyzed using quantitative real-time reverse transcription-PCR method (qRT-PCR). *p* < 0.05 was considered statistically significant and represented by different letters appeared above each bar.

*SmCHSs* exhibited differential expression in roots, stems, leaves and flowers of *S. miltiorrhiza* (Figure 3). *SmCHS1*, *SmCHS4* and *SmCHS5* were predominantly expressed in flowers, whereas *SmCHS7* and *SmCHS8* were predominantly expressed in roots and stems, respectively. Both *SmCHS2* and *SmCHS6*, two *ASCLs*, showed the highest expression levels in flowers. Based on the anther-specific expression of other plant *ASCLs* [28], we speculated that high *SmCHS2* and *SmCHS6* transcripts in flowers probably originate from anthers. The expression pattern of *SmCHS3* was similar in flowers, stems and roots. The expression level in leaves was very low. Differential expression of *CHSs* was also observed in other plant species, such as *I. purpurea* [22]. This indicates that different *SmCHSs* may have different physiological functions in a plant.

### 2.3. SmCHI1–SmCHI4

CHIs usually exist as a multigene family and can be divided into four types, including type I–IV in previous studies [29,30,31,32]. Type I CHIs are ubiquitous in vascular plants. They catalyze the conversion of 6′-hydroxychalcone (naringenin chalcone) to (2S)-5-hydroxyflavanone. Type II CHIs usually exist in leguminous plants. They not only play the role of type I CHIs, but also convert 6′-deoxychalcone into 5-deoxyflavonoid. Type III CHIs are fatty acid-binding proteins (FAPs) widely distributed in land plants and green algae. FAPs affect the biosynthesis of fatty acids in plant cells and its storage in developing embryos [29]. Type IV CHIs are CHI-like proteins (CHILs) only found in land plants. CHILs act as the enhancer of flavonoid production (EFP) to promote the biosynthesis of flavonoids and flower pigmentation [30,31]. Generally, CHI proteins of the same type show around 70% or more identities, whereas CHIs belonging to different types show less than 50% identities [32].

Since the first identification from cell cultures of bean CHIs (*Phaseolus vulgaris*) [33], they have been cloned and characterized from various higher plant species, such as *A. thaliana* [31], *Zea mays* [34], *Lotus japonicas* [32], and *Solanum lycopersicum* [35]. *A. thaliana* has five CHIs, including a Type I CHI (AtCHI), three Type III CHIs (AtFAP1, AtFAP2 and AtFAP3), and a type IV CHI (AtCHIL) [31]. From the genome of *S. miltiorrhiza*, we identified four genes encoding SmCHIs. All of them contain three introns (Supplementary Figure S1). It is consistent with *CHI genes* from other plant species [32]. The deduced proteins of all four SmCHIs possess the conserved domain, known as the chalcone domain (pfam02431) (Supplementary Figure S2), and share high sequence identities with CHI or CHI-like proteins from other plant species. SmCHI1, SmCHI2 and SmCHI4 have more than 76% identities with type I CHIs from *Perilla frutescens* (BAG14301), *Agastache rugosa* (AFL72080), and *Scutellaria baicalensis* (ADQ13184.1). SmCHI3 shares over 68% identity with type IV CHIs from *A. thaliana* (AT5g05270) [31] and *Ipomoea nil* (BAO58578.1) [30]. Protein sequence alignments of SmCHI1–SmCHI4 to *M. sativa* MsCHI and AtCHI and AtCHIL showed that SmCHI1, SmCHI2 and SmCHI4 shared more conserved amino acid residues with MsCHI and AtCHI than other species in the database [7,29] (Supplementary Figure S4 online). The critical catalytic residues of type I and type II CHIs, including Arg36, Thr48, Tyr106, Asn113, and Thr/Ser190 (numbers refer to MsCHI), were highly conserved among SmCHI1, SmCHI2, SmCHI4, MsCHI, and AtCHI. However, many of these residues were substituted in SmCHI3 and AtCHIL. It indicates that SmCHI1, SmCHI2 and SmCHI4 are type I CHIs, whereas SmCHI3 belongs to type IV.

**Figure 4:**
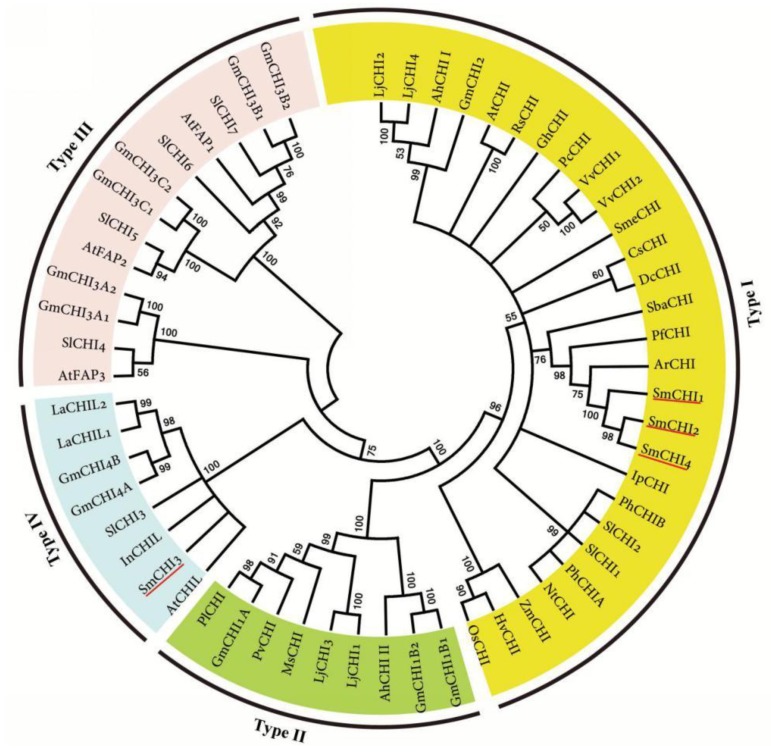
The phylogenetic relationship of CHI proteins. The numbers represent the bootstrap values. These CHI protein sequences used for phlogenetic analysis were retrieved from NCBI and their accession numbers are listed in Supplementary Table S5.

Phylogenytic analysis of SmCHIs and CHIs from other plant species showed that plant CHIs are resolved into four distinct clades (Types I-IV) corresponding to protein sequence and function (Figure 4). This is consistent with previous studies [29,30,31,32]. SmCHI1, SmCHI2 and SmCHI4 cluster with CHIs from other characterized type I CHI, such as AtCHI [31], *Z. mays* ZmCHI [34], *S. lycopersicum* SlCHI1 and SlCHI2 [35]. SmCHI3 is included in the clade with type IV CHIs, such as AtCHIL [31], *I. nil* InCHIL [30], and *Lupinus angustifolius* LaCHIL1 and LaCHIL2 [36]. It is consistent with the results from sequence identity comparison and conserved amino acid residue analysis, implying the capability of SmCHI1, SmCHI2 and SmCHI4 in the cyclization of bicyclic chalcones to tricyclic (S) flavanones and the involvement of SmCHI3 in enhancing flavonoid biosynthesis.

qRT-PCR analysis of *SmCHI* gene expression in flowers, leaves, stems and roots of *S. miltiorrhiza* showed that all of them had the highest expression level in flowers (Figure 3). Similar results were also observed for *ArCHI* from the related plant species, *Agastache rugosa* [37]. It is consistent with the fact that flowers usually contain abundant anthocyanins and further suggests the involvement of *SmCHIs* in flavonoid biosynthesis.

### 2.4. SmFNSII, SmF3′5′H and SmF3′Hs

The enzymes FNSII, F3′5′H and F3′H are members of the cytochrome P450-dependent monooxygenase (P450) superfamily, a large class of heme-containing and membrane-localized monooxygenases usually using NADPH and molecular oxygen as co-substrates to catalyze the hydroxylation reactions [10]. The P450 genes involved in flavonoid biosynthesis have been cloned and characterized in various plant species, such as *S. baicalensis* [38], *V. vinifera* [39], and *Camellia sinensis* [40]. From *S. miltiorrhiza*, one *SmFNSII*, one *SmF3′5′H* and six *F3′Hs* were identified (Table 1). SmFNSII, SmF3′5′H and SmF3′H1–SmF3′H2 proteins show high sequence identities (≥74%) with *S. baicalensis* FNSII (AMW91728), *Antirrhinum kelloggii* F3′5′H (BAJ16329), and *P. frutescens* F3′H (BAB59005), respectively. SmF3′H3–SmF3′H6 have high identities with F3′H-likes from various plants, such as *Sesamum indicum* (XP_011095827) and *Erythranthe guttata* (XP_012854737).

The identified protein sequences contain the p450 domain (pfam00067) (Supplementary Figure S2) and include the proline-rich hinge region, the oxygen-binding pocket, the E-R-R triade, and the heme-binding domain (Supplementary Figures S5–S7). The proline-rich hinge region acts as a “hinge” and is indispensable for optimal orientation of the P450 enzymes to membrane [41]. The oxygen-binding pocket motif forms a threonine-containing pocket to bind oxygen molecules [42]. The E-R-R triade, which consists of the E and R from the ExxR consensus sequence and the R from the “PERF” consensus sequence, is involved in locking the heme pockets into position and to assure stabilization of the conserved core structure [43]. The heme-binding domain FxxGxxxCxG is critical for P450 to bind heme. Its cysteine (C) is invariantly conserved, whereas the phenylalanine (F) and two glycines (G) are generally, but not always conserved [44]. The enzyme sequences SmF3′H1 and SmF3′H2, but not SmF3′H3–SmF3′H6, contain three typical F3′H-specific conserved motifs, including VVVAAS, GGEK, and VDVKG [45] (Supplementary Figure S6). These results suggest that SmFNSII, SmF3′5′H and SmF3′H1–SmF3′H6 are members of the P450 superfamily. Among them, SmF3′H1 and SmF3′H2 are typical F3′Hs, whereas the function of SmF3′H3–SmF3′H6 remains to be elucidated.

**Figure 5:**
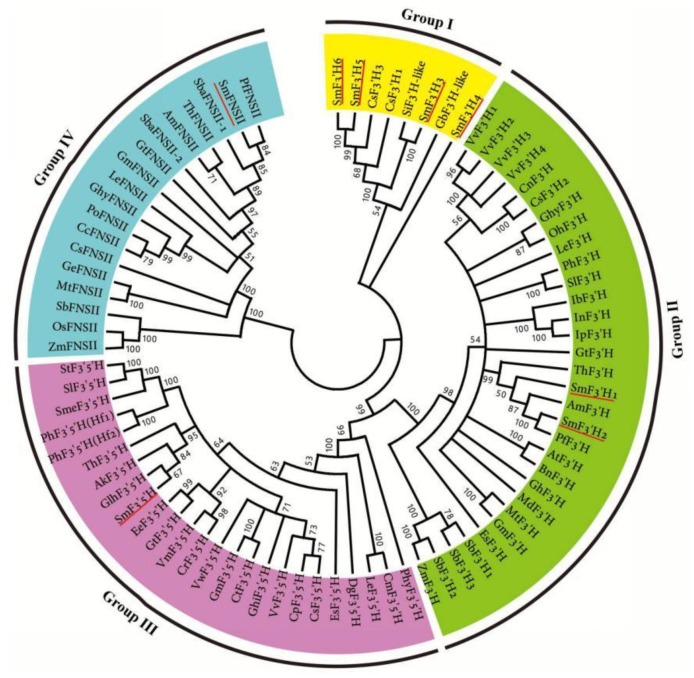
The phylogenetic relationship of FNSII, F3′5′H and F3′H proteins. The amino acid sequences of flavonoid biosynthesis-related P450s, including FNSII, F3′5′H and F3′H were obtained from NCBI under the accession numbers listed in Supplementary Table S6.

To investigate the phylogenetic relationship of FNSII, F3′5′H and F3′H, a phylogenetic tree was constructed (Figure 5). SmFNSII clusters with known FNSIIs, of which GeFNSII, MtFNSII, SbFNSII, OsFNSII and ZmFNSII exhibit 2-hydroxylation activity and catalyze the biosynthesis of the 2-hydroxyflavanone intermediate, a substrate of flavone C-glycoside biosynthesis [46]. Various other FNSIIs, such as two Labiatae FNSIIs, including PfFNSII [47] and SbaFNSII-1 [38], directly convert flavanones to flavones, which are further transformed into flavone O-glycosides. SmFNSII groups with high bootstrap support with PfFNSII and SbaFNSII-1 (Figure 5). It indicates that SmFNSII can catalyze the conversion of flavanones to flavones. SmF3′5′H clusters with the characterized F3′5′Hs from *A. kelloggii* [48], *S. lycopersicum* [49], *V. vinifera* [39], and *C. sinensis* [40]. SmF3′H1 and SmF3′H2 cluster with F3′Hs from *P. frutescens* [47], *Torenia hybrida* [50], and other typical F3′Hs. SmF3′H3–SmF3′H6 are separated from SmF3′H1 and SmF3′H2 and cluster with F3′H-likes from other plants, of which CsF3′H1 and CsF3′H3 are key enzymes closely related with the ratio of dihydroxylated to trihydroxylated catechins in *C. sinensis* [51]. It is consistent with the results from phylogenetic relationship analysis of CsF3′H1–CsF3′H3 [45], and indicate that the function of SmF3′H3–SmF3′H6 is different from typical F3′Hs.

The expression of *SmFNSII*, *SmF3′5′H* and *SmF3′H1*–*SmF3′H6* in roots, stems, leaves and flowers of *S. miltiorrhiza* was analyzed using the qRT-PCR method (Figure 3). *SmFNSII* showed the highest expression in flowers. The expression pattern of *SmFNSII* is similar to *Gentiana triflora FNSII* showing preferential expression in petals compared with leaves and stems [52]. *SmF3′5′H* was predominantly expressed in flowers. It has been shown that *F3′5′H* plays indispensable roles in the biosynthesis of delphinidin-based anthocyanins, which usually make flower petals violet or blue [10,48]. Since the flowers of *S. miltiorrhiza* (line 99–3) are violet, we speculate that *SmF3′5′H* play important roles in the formation of flower pigments. *SmF3′Hs* exhibited differential expression patterns. *SmF3′H1* had similar expression levels in all four tissues analyzed. *SmF3′H2* and *SmF3′H4* showed the highest expression in flowers. *SmF3′H3* was predominantly expressed in roots. *SmF3′H5* and *SmF3′H6* showed similar expression in roots, stems and flowers. Expression was relatively low in leaves. Differential expression was also observed for functional distinct groups in *Sorghum bicolor F3′Hs* [53]. This indicates functional divergence of *SmF3′Hs* in *S. miltiorrhiza*.

### 2.5. SmF3H, SmFLS, and SmANS

F3H, FLS, and ANS belong to the 2-oxoglutarate dependent dioxygenase (2-ODD) superfamily. 2-ODDs are a class of iron-containing and cytosol-localized non-heme oxygenases. They require ferrous iron Fe (II) as the active site cofactor and 2-oxoglutarate (2OG) and molecular oxygen as the co-substrates for catalyzing the oxidation of an organic substrate [9]. F3H, FLS, and ANS are all involved in the oxidative modifications of the C-ring of the flavonoid backbone [9]. F3H acts in the upstream step towards the biosynthesis of flavonols, anthocyanins and PAs. FLS catalyzes the specific downstream step towards flavonol biosynthesis, whereas ANS catalyzes the specific downstream step towards the biosynthesis of anthocyanins and PAs. Genes encoding F3H, FLS, and ANS have been studied in various plant species, such as *A. thaliana* [54,55,56], *Petunia hybrida* [57,58,59], and *Punica granatum* [60].

*S. miltiorrhiza* has one ANS, two F3Hs, and two FLSs (Table 1). All of them contain the DIOX_N domain (pfam14226) conserved in the N terminal region of 2-ODDs and the 2OG-FeII_Oxy domain (pfam03171) highly conserved in the C terminus (Supplementary Figure S2). Genomic structure analysis showed that *SmF3H1*, *SmF3H2* and *SmFLS2* contained three exons, whereas *SmFLS1* and *SmANS* included two (Supplementary Figure S1). The deduced protein sequences of SmF3H1 and SmF3H2 have 76% and 73% identity with *P. hybrida* PhF3H, respectively [57]. SmFLS1 and SmFLS2 show 59% and 74% identities with PhFLS [58], respectively. SmANS shares 81% identities with PhANS [59].

**Figure 6:**
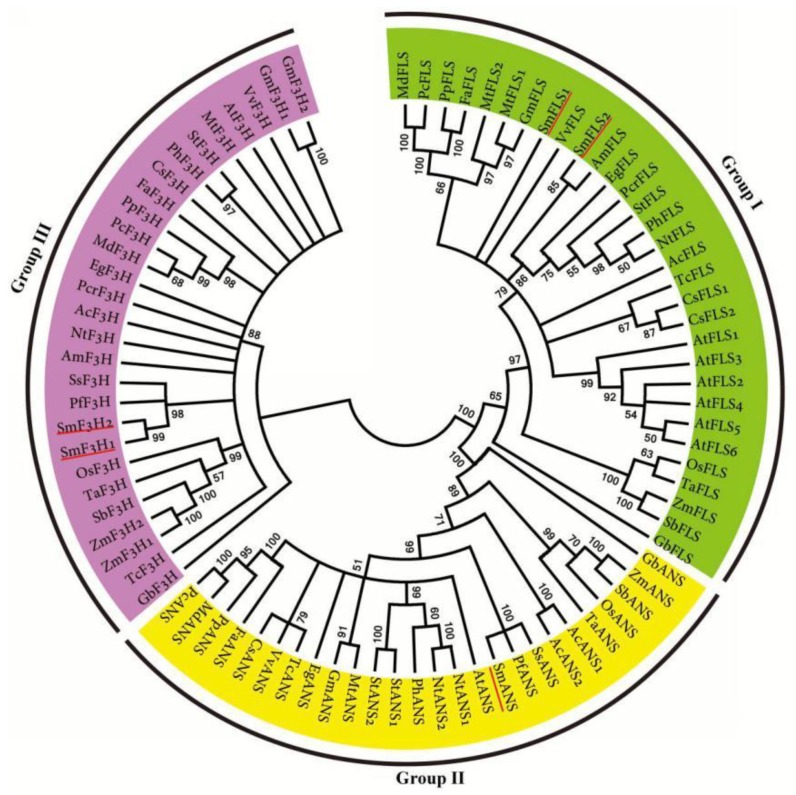
The phylogenetic relationship of FLS, F3H and ANS proteins. The amino acid sequences of flavonoid biosynthesis-related 2-ODDs, including FLS, F3H and ANS, were obtained from NCBI under the accession numbers listed in Supplementary Table S7.

Based on the crystal structure of *A. thaliana* ANS^54^, His-232, His-288 and Asp-234 (numbering refers to AtANS) in the conserved H-x-D-xn-H motif are required for binding FeII iron. Tyr-217, Arg-298 and Ser-300 in the conserved R-x-S motif are involved in binding 2OG [54,61]. These six critical residues forming two motifs are highly conserved in most 2-ODDs. Consistently, all of the identified *S. miltiorrhiza* 2-ODDs, including SmF3H1, SmF3H2, SmFLS1, SmFLS2, and SmANS, contain the six critical residues (Supplementary Figures S8–S10). In addition, the substrate-binding residues found in AtANS, AtF3H, and AtFLS are conserved in SmANS, SmF3Hs and SmFLSs, respectively [54,55,56]. Seven highly conserved residues (Met-105, Ile-114, Val-115, Ile-130, Asp-194, Leu-214 and Lys-215) with critical roles in determining the activity of F3Hs exist in SmF3H1 and SmF3H2 [55] (Supplementary Figure S8). These conserved residues suggest the catalytic role of SmANS, SmF3Hs, and SmFLSs. The relationships among SmF3Hs, SmFLSs, SmANS and their homologous from other plants were analyzed using a phylogenetic tree constructed by the neighbor-joining method. F3Hs, FLSs and ANSs are clearly separated into three clades (Figure 6). It is consistent with the 2-ODD phylogenetic tree constructed by Tohge et al. [62]. *SmF3H1* and *SmF3H2* had the highest expression level in flowers and the least in roots (Figure 3). *SmFLS1* is mainly expressed in flowers, whereas the expression of *SmFLS2* showed the highest levels in flowers and leaves, less in stems, and and the lowest in roots. This indicates that the two *SmFLSs* play distinct physiological roles in *S. miltiorrhiza*. *SmANS* is predominantly expressed in the anthocyanin-abundant flowers. This is in accordance with the indispensable role of ANS in anthocyanin biosynthesis [60].

### 2.6. SmDFR

DFR is a nicotinamide adenine dinucleotide phosphate (NADPH)-dependent oxidoreductase and belongs to the short-chain dehydrogenase/reductase (SDR) superfamily [11]. It was first reported in *Z. mays* [63]. So far, DFR has been investigated in various species, such as *V. vinifera* [11], *Lotus japonicas* [64] and *Brassica rapa* [65]. Through genome-wide analysis, we identified a *SmDFR* gene in *S. miltiorrhiza*. It contains six exons (Supplementary Figure S1) as *DFRs* in other plant species, such as *L. japonicas* [64] and *B. rapa* [65]. Sequence feature of *SmDFR* is shown in Table 1. SmDFR has 89%, 83% and 84% identity with DFRs from *Solenostemon scutellarioides* (ABP57077.1), *P. frutescens* (BAA19658.1), and *Erythranthe lewisii* (AHJ80979.1), respectively. The deduced SmDFR protein contains the conserved epimerase domain found in other plant DFRs [65] (Supplementary Figure S2). 

Amino acid sequence alignment of SmDFR and DFRs from *G. hybrida*, *P. hybrida*, *A. thaliana*, *V. vinifera* and *M. domestica* showed that plant DFRs were highly conserved in the catalytic core (Supplementary Figure S11). All of them contain the NADPH-binding motif [64], the conserved catalytic triad site (Ser-129, Tyr-164, Lys-168) revealed in the crystal structure of *V. vinifera* DFR, and the substrate-binding region responsible for substrate specificity [11]. It has been shown that DFRs with the Asn (N) residue at the corresponding position of the 134th of *G. hybrida* DFR are able to utilize all three dihydroflavonols, including dihydrokaempferol (DHK), dihydroquercetin (DHQ), and dihydromyricetin (DHM) as substrates, whereas mutation of the Asn (N) residue to Asp (D) results in lacking the ability to accept DHK as the substrate to produce leucopelargonidin efficiently in *Petunia hybrida* and *Cymbidium hybrida* DFRs [66,67,68]. SmDFR possesses the Asn (N) residue, indicating it could use all three dihydroflavonols as substrates.

**Figure 7:**
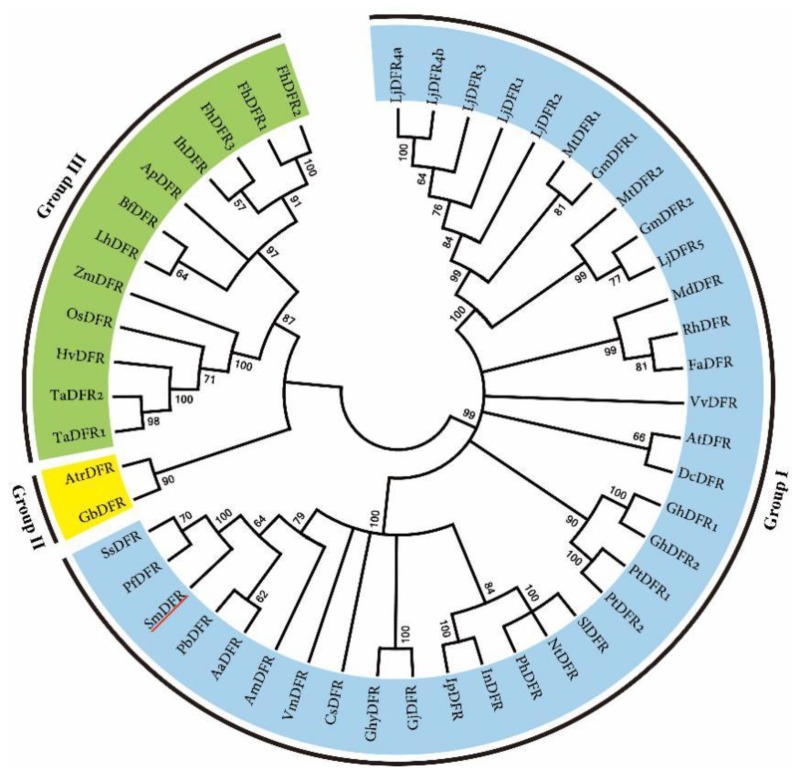
The phylogenetic relationship of DFR proteins. DFR amino acid sequences from various plant species were obtained from NCBI under the accession numbers listed in Supplementary Table S8 online.

Phylogenetic analysis showed that DFRs from monocots clustered in one clade, while DFRs from dicots clustered in the other. DFRs from *Ginkgo biloba* (gymnosperm) and the earliest diverging lineage in the clade of angiosperms, *Amborella trichopoda* [69], clustered with monocot DFRs (Figure 7). Consistent with angiosperm phylogeny, SmDFR located in the dicot clade showed a close relationship with other DFRs from related orders, such as Lamiales, Solanales, Asterales, and Ericales [69]. *SmDFR* was predominantly expressed in flowers (Figure 3). It is consistent with its vital role in anthocyanin biosynthesis.

### 2.7. Responses of Flavonoid Biosynthesis-Related Genes to Exogenous MeJA

Methyl jasmonate (MeJA) is a signaling molecule involved in plant growth, development and defense, particularly in response to insect and pathogen attack, wounding and disease [3,70,71,72]. MeJA can induce flavonoid biosynthetic gene expression and has been found to enhance flavonoid accumulation in *Rubus* sp. [73], *V. vinifera* [74], and *Coleus forskohlii* [75]. It has also been used as an elicitor to regulate the transcription of genes involved in phenolic acid and tanshinone biosynthesis in *S. miltiorrhiza* [13,76,77]. However, the effects of MeJA on flavonoid biosynthetic genes of *S. miltiorrhiza* were unknown. We analyzed the expression of the identified 26 genes in *S. miltiorrhiza* roots and leaves treated with exogenous MeJA. 

**Figure 8:**
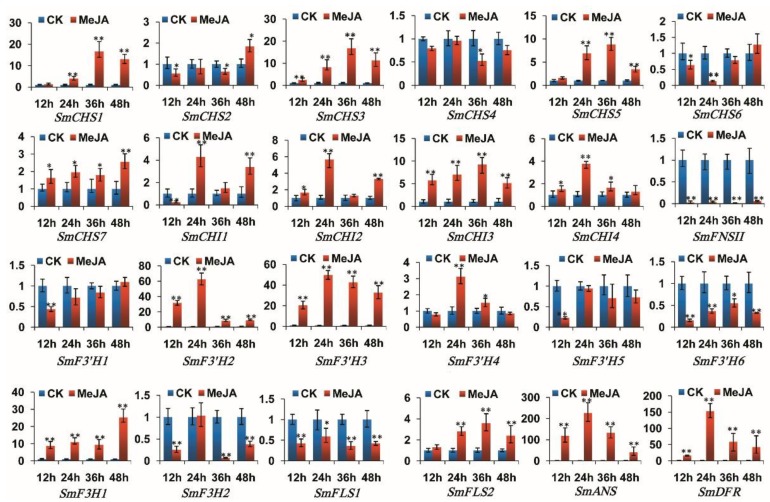
Expression of flavonoid biosynthesis-related genes in roots of *S. miltiorrhiza* treated with MeJA for 12 h, 24 h, 36 h and 48 h. The levels of transcripts were analyzed using the qRT-PCR method. *p* < 0.05 (*) and *p* < 0.01 (**) were considered statistically significant and extremely significant, respectively.

**Figure 9:**
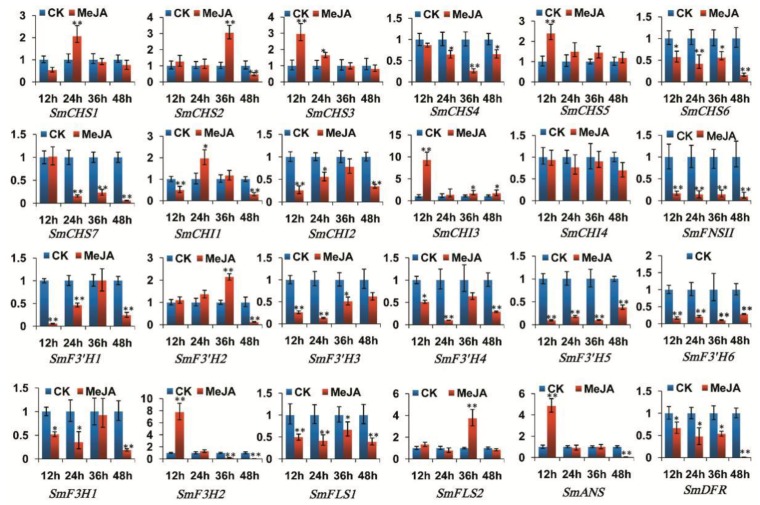
Expression of flavonoid biosynthesis-related genes in leaves of *S. miltiorrhiza* treated with MeJA for 12 h, 24 h, 36 h and 48 h. qRT-PCR method were used. *p* < 0.05 (*) and *p* < 0.01 (**) were considered statistically significant and extremely significant, respectively.

In MeJA-treated S. miltiorrhiza roots, SmCHS1, SmCHS3, SmCHS5, SmCHS7, SmCHI2–SmCHI4, SmF3′H2–SmF3′H4, SmF3H1, SmFLS2, SmDFR and SmANS were significantly up-regulated, whereas SmCHS4, SmCHS6, SmFNSII, SmF3′H1, SmF3′H5, SmF3′H6, SmF3H2 and SmFLS1 were significantly down-regulated in at least some time-point of MeJA treatment (Figure 8). In MeJA-treated S. miltiorrhiza leaves, SmCHS1, SmCHS3, SmCHS5, SmCHI3, SmF3H2 and SmFLS2 were up-regulated, whereas SmCHS4, SmCHS6, SmCHS7, SmCHI2, SmF3′H1, SmF3′H3–SmF3′H6, SmF3H1, SmFLS1, SmFNSII and SmDFR were down-regulated at different levels (Figure 9). 

In addition, the expression levels of *SmCHS2* and *SmCHI1* in roots and *SmCHS2*, *SmCHI1*, *SmF3′H2* and *SmANS* in leaves were increased or decreased at different time-points of MeJA treatment. No significant changes were observed for the expression of *SmCHI4* in leaves. The results indicate that *S. miltiorrhiza* flavonoid biosynthesis-related genes were MeJA-responsive and the responses were in a tissue- and time-specific manner. It is consistent with the results from other plant species showing the regulation of MeJA in flavonoid (particularly anthocyanin) accumulation [78] and the stimulation of anthocyanin biosynthetic genes, including *CHS* [74,79], *CHI* [80], *F3H* [80,81], *F3′H* [80], *DFR* [78] and *ANS* [78].The expression levels of *SmCHS1*, *SmCHS3*, *SmCHS5*, *SmCHI3*, *SmCHI4*, *SmF3′H2*, *SmF3′H3*, *SmF3′H4*, *SmFLS2*, *SmANS*, and *SmDFR* in roots reached the peak at the time-point of 24 h or 36 h treatment, and then gradually decreased. Similar results were observed for *CHS* expression in hairy roots of *Scutellaria viscidula* [79]. This indicates that these genes can cooperate with each other in response to MeJA treatment. In addition, some genes from the same gene family showed differential responses to MeJA treatment. For example, *SmF3H1* was significantly up-regulated in roots and down-regulated in leaves, whereas its paralog, *SmF3H2*, was down-regulated in roots and up-regulated first and then down-regulated in leaves. Similarly, two members of the *FNSII* family from *S. baicalensis*, including *SbaFNSII-1* and *SbaFNSII-2*, were differentially expressed in hairy roots of *S. baicalensis* treated with MeJA. *SbaFNSII-1*, which is involved the production of normal 4′-hydroxyflavones, showed no obvious change after MeJA treatment. However, *SbaFNSII-2* involved in 4′-deoxyflavanone biosynthesis was significantly increased after treatment [38]. It indicates that different flavonoid biosynthetic gene members of a family may respond differentially to external stimuli and play distinct roles in different tissues.

### 2.8. Gene Candidates Encoding Flavonoid Biosynthetic Enzymes

The twenty six identified genes are members of the *CHS*, *CHI*, *FNSII*, *F3H*, *F3′H*, *F3′5′H*, *FLS*, *ANS* and *DFR* families, of which *FNSII*, *F3′5′H*, *ANS* and *DFR* each has only one gene in *S. miltiorrhiza*. Thus, *SmFNSII*, *SmF3′5′H*, *SmANS* and *SmDFR* appear to encode flavonoid biosynthetic enzymes. Based on sequence identity comparison, conserved amino acid residue analysis, gene expression patterns and phylogenetic analysis, we proposed that, among the eight *SmCHSs*, *SmCHS1* is the most likely one involved in flavonoid biosynthesis. Other seven *SmCHSs* might play similar function with their homologs in the same group which has been discussed in the phylogenetic analysis of CHS. Among the four *SmCHIs*, *SmCHI1*, *SmCHI2* and *SmCHI4* probably encode enzymes catalyzing the conversion of chalcone to flavanone, whereas *SmCHI3*, similar to *AtCHIL*, acts as an enhancer of flavonoid biosynthetic pathway. *SmF3H1* and *SmF3H2* encode typical F3Hs catalyzing the 3-hydroxylation of (2*S*)-flavanone to (2*R*,3*R*)-dihydroflavonols. These genes could play different physiological roles, since they show distinct expression patterns in plant tissues and in response to MeJA treatment. Similar to *SmF3H1* and *SmF3H2*, *SmF3′H1* and *SmF3′H2* encode flavonoid biosynthetic enzymes catalyzing the 3′-hydroxylation of the B ring of naringenin or dihydrokaempferol [10], although they are expressed differentially in different tissue and in response to MeJA treatment. SmF3′H3-SmF3′H6 might play different roles from common F3′Hs, since their significant divergence in protein sequence features and phylogenetic relationship. SmFLS1 and SmFLS2 are typical FLSs catalyzing the desaturation of dihydroflavonol to the most abundant and widespread flavonoid subgroup, flavonols. Taken together, a total of fourteen genes are most likely to encode common flavonoid biosynthetic enzymes.

Among those fourteen genes, *SmCHS1*, *SmCHI1*, *SmCHI2*, *SmCHI4*, *SmF3H1*, *SmF3H2*, *SmF3′H1*, *SmF3′H2*, and SmF3′5′H are early biosynthetic genes (EBGs) of the flavonoid pathway involved in the biosynthesis of common intermediates. *SmFLS1* and *SmFLS2*, *SmFNSII*, *SmANS* and *Sm*DFR are late biosynthetic genes (LBGs) involved in the production of specific flavonoid subgroups, such as flavonols, flavones and anthocyanins. The roles of the other twelve of the twenty six identified genes remain to be elucidated. Some of them, such as *SmCHI3*, may also be involved in flavonoid biosynthesis, although they do not encode typical flavonoid biosynthetic enzymes.

## 3. Materials and Methods

### 3.1. Plant Materials and MeJA Treatment

*Salvia miltiorrhiza* (line 99–3) plants with the whole genome sequence available [16] were cultivated in the field nursery of the Institute of Medicinal Plant Development (IMPLAD). Fresh roots, stems, leaves and flowers were collected from two-year-old *S. miltiorrhiza* plants in July of 2016 when the aerial parts were growing vigorously. Plantlets and media used for MeJA treatment were prepared as described previously [13]. Plantlets were treated with MeJA (200 μM) for 12 h, 24 h, 36 h and 48 h, respectively. Plantlets treated with carrier solution were used as controls. Similar sizes of leaves and roots were collected from MeJA-treated and control plantlets at same time. All samples were stored in liquid nitrogen until use. Three independent biological replicates were performed.

### 3.2. Genome-Wide Survey and Gene Prediction

The deduced protein sequences of fourteen flavonoid biosynthesis-related genes, including *C. sinensis CsF3′5′H*, *Glycine max GmF3′5′H*, *Arabidopsis thaliana AtCHS*, *AtCHI*, *AtF3H*, *AtFLS1*, *AtFLS2*, *AtFLS3*, *AtFLS4*, *AtFLS5*, *AtFLS6*, *AtF3′H*, *AtDFR* and *AtANS*, were downloaded from the National Center for Biotechnology Information (NCBI) database (Supplementary Table S1). The tblastn algorithm was used to search the *S. miltiorrhiza* line 99–3 genome assembly for homologues of the downloaded flavonoid biosynthetic enzymes [16,18]. A cut-off e-value of 1e–10 was applied. Gene models of all retrieved sequences were predicted on the GenScan web server (http://genes.mit.edu/GENSCAN.html) [82]. The predicted gene models were further examined by BLAST analysis against the non-redundant protein sequence (nr) database (http://www.ncbi.nlm.nih.gov/BLAST) using the BLASTx algorithm with default parameters [18]. Gene models were manually corrected by careful comparison of the predicted gene model with genes identified in other plant species.

### 3.3. RNA extraction, cDNA Cloning and Quantitative Real-Time Reverse Transcription-PCR (qRT-PCR)

Total RNA was extracted from *S. miltiorrhiza* tissues using the plant total RNA isolation kit (Aidlab, Beijing, China) as per the manufacturer’s protocol. Genomic DNA contamination was eliminated during the course of extraction. The integrity of total RNA was analyzed on a 1.2% agarose gel. RNA quantity was determined using a NanoDrop 2000C spectro-photometer (Thermo Scientific, Waltham, MA, USA). Reverse transcription was performed on total RNA using the Superscript III reverse transcriptase (Invitrogen, Waltham, MA, USA). Full length CDSs were amplified by PCR using gene specific primers listed in Supplementary Table S2. PCR products with expected size were gel-purified, cloned into pMD18T-vector and sequenced. qRT-PCRs were performed as described previously [83]. Gene-specific primers are listed in Supplementary Table S3. The length of amplicons was between 100 and 350 bp. SmUBQ10 was used as a reference gene as described previously [83]. For tissue specific expression analysis of genes in flowers, leaves, stems and roots, the transcript level in stem was arbitrarily set to 1 and the levels in other tissues were given relative to this. For the the expression analysis of MeJA treatment, the transcript level in tissues without MeJA treatment was arbitrarily set to 1 and the levels in tissues of MeJA-treated plantlets were given relative to this. All samples were detected as triplicates in three independent biological replicates. The data from gene specific amplification were analyzed as described previously [83]. Error bars represent standard deviations of mean value from three biological and three technical replicates. Analysis of variance (ANOVA) was calculated using SPSS. 

### 3.4. Sequence Feature and Phylogenetic Analysis

Intron/exon structure was determined by pairwise alignment of the full-length cDNA sequences and the corresponding genomic sequences on the Gene Structure Display Server 2.0 (http://gsds.cbi.pku.edu.cn) [84]. The theoretical isoelectric point (pI) and molecular weight (Mw) were predicted using the Compute pI/MW tool on the ExPASy server (http://web.expasy.org/compute_pi/) [85]. Conserved domains were searched against the Pfam protein family database (http://pfam.xfam.org/) [86]. Phylogenetic trees of full length protein sequences were constructed by the neighbor joining (NJ) method with 1000 bootstrap replicates [32,40,65] using Molecular Evolutionary Genetics Analysis Version 7.0 (MEGA version 7.0, http://www.megasoftware.net) [87]. For each analysis, only nodes supported by bootstrap values greater than 50% are shown. The numbers on the branches represent bootstrap support in percentages. The genes from *S. miltiorrhiza* were highlighted by red underline. Proteins used for phylogenetic analysis were listed in Supplementary Tables S4–S8. 

## 4. Conclusions

Systematic analysis of the *S. miltiorrhiza* genome and subsequent molecular cloning allows us to identify twenty six flavonoid biosynthesis-related genes, including *SmCHS1–SmCHS8*, *SmCHI1–SmCHI4*, *SmFNSII*, *SmF3′5′H*, *SmF3′H1–SmF3′H6*, *SmF3H1–SmF3H2*, *SmFLS1–SmFLS2*, *SmANS* and SmDFR. Among them, twenty are the first to be described. The deduced protein sequences share various structural similarities with the corresponding proteins from other plant species and have close phylogenetic relationships with their homologs in other plant species. At present, the molecular evolution of flavonoid biosynthesis-related genes, such as *CHS* and *CHI*, has been studied in plants. Generally speaking, these genes are derived from gene duplication. Subsequent mutations allow them to gain new functions [88,89]. In this study, we found multiple members in *CHS* and *CHI* families. Duplication and mutation could also occur for these genes. In addition, the majority of the identified genes showed high expression levels in the aerial part, which is consistent with the accumulation of flavonoids in stems, leaves and flowers of *S. miltiorrhiza* [90]. Under exogenous MeJA treatment, different members of the *SmCHS*, *SmCHI*, *SmF3H*, *SmF3′H* and *SmFLS* gene families showed distinct spatial and temporal expression patterns, indicating different biochemical and/or physiological functions. Among the twenty six identified genes, fourteen probably encode flavonoid biosynthetic enzymes, whereas the other twelve need to be further investigated. For example, CRISPR/Cas9 mutagenesis of identified genes in vivo would be helpful to understand the distinct role of these genes in *S. miltiorrhiza*. Enzyme assays in vitro will be useful for functional elucidation of distinct genes. Taken together, our results provide insights into the flavonoid biosynthesis enzyme genes in *S. miltiorrhiza*, an important medicinal plant with great economic and medicinal value. 

## Figures and Tables

**Table 1:** Sequence features of flavonoid biosynthesis-related genes in *S. miltiorrhiza*.

Gene Name	ORF (bp) ^1^	AA Len ^2^	Mw (Da) ^3^	pI ^4^	Accession Number ^5^
SmCHS1	1173	390	42,574.02	5.98	MH447681
SmCHS2	1161	386	42,388.73	5.74	MH447682
SmCHS3	1179	392	42,796.26	5.66	MH447683
SmCHS4	1173	390	41,868.1	5.97	MH447684
SmCHS5	1176	391	42,232.43	5.56	MH447685
SmCHS6	1161	386	42,242.52	5.61	MH447686
SmCHS7	1170	389	42,088.31	5.77	MH447687
SmCHS8	1173	390	42,265.73	6.48	MH447688
SmCHI1	678	225	23,983.43	4.9	MH447677
SmCHI2	678	225	23,920.41	5.08	MH447680
SmCHI3	615	204	22,769.91	4.9	MH447678
SmCHI4	678	225	24,003.59	5.23	MH447679
SmF3H1	1050	349	39,405.87	5.46	MH447666
SmF3H2	1056	351	39,624.12	5.45	MH447667
SmF3′5′H	1551	516	57,449.89	8.62	MH447665
SmF3′H1	1536	511	56,247.24	8.16	MH447668
SmF3′H2	1545	514	56,556.4	7.31	MH447669
SmF3′H3	1560	519	59,412.96	7.73	MH447670
SmF3′H4	1557	518	57,620.86	8.18	MH447671
SmF3′H5	1530	509	57,819.46	8.84	MH447672
SmF3′H6	1530	509	58,162.86	8.94	MH447673
SmFLS1	972	323	36,628.78	5.58	MH447674
SmFLS2	1008	335	37,997.42	5.4	MH447675
SmFNSⅡ	1533	510	57,339.73	8.59	MH447676
SmDFR	1143	380	42,568.53	5.25	MH447664
SmANS	1110	369	41,574.54	5.33	MH447663

^1^.ORF, open reading frame; ^2^. AA len, the number of amino acid residues; ^3^. Mw, molecular weight; ^4^. *pI*, theoretical isoelectric point; ^5^. Accession number: GenBank accession numbers for the nucleotide sequences of all those genes.

## Supplementary Materials

The following are available online at http://www.mdpi.com/1420-3049/23/6/1467/s1. Table S1: Sequence features of flavonoid metabolism pathway genes in *Arabidopsis thaliana and other plants;* Table S2: Primers used for full-length coding region cloning; Table S3: Primers used for qRT-PCR; Table S4: The proteins used in the phylogenetic tree of CHS; Table S5: The proteins used in the phylogenetic tree of CHI; Table S6: The proteins used in the phylogenetic tree of FNSII, F3′5′H and F3′H; Table S7: The proteins used in the phylogenetic tree of F3H, FLS and ANS; Table S8: The proteins used in the phylogenetic tree of DFR; Figure S1: Exon/intron Structures of flavonoid biosynthesis related genes; Figure S2: Conserved domains in flavonoid biosynthesis related proteins in *S. miltiorrhiza*; Figure S3: Amino acid sequence alignment of SmCHS1–SmCHS8 against *Medicago sativa* MsCHS2, and *Arabidopsis thaliana* AtCHS; Figure S4: Amino acid sequence alignment of SmCHI1–SmCHI4 against *Medicago sativa* MsCHI, *Arabidopsis thaliana* AtCHI and AtCHIL; Figure S5: Comparison of SmFNSII proteins with other plant FNSII proteins; Figure S6: Amino acid sequence alignment of SmF3′H1–SmF3′H6 against *Arabidopsis thaliana* AtF3′H from and Petunia hybrid PhF3′H; Figure S7: Amino acid sequence alignment of SmF3′5′H against *Glycine max* GmF3′5′H, *Petunia hybrid* PhF3′5′H (Hf1) and PhF3′5′H (Hf2), *Solanum lycopersicum* SlF3′5′H, *Vitis vinifera* VvF3′5′H; Figure S8: Amino acid sequence alignment of SmF3H1 and SmF3H2 against other plant F3Hs; Figure S9: Amino acid sequence alignment of SmFLS1 and SmFLS2 against other plant FLSs; Figure S10: Amino acid sequence alignment of SmANS against other plant ANSs; Figure S11: Amino acid sequence alignment of SmDFR against other plant DFRs.
