# Supplementary material for: Identification and Characterization of Flavonoid Biosynthetic Enzyme Genes in Salvia miltiorrhiza (Lamiaceae)
Source: Molecules. 2018 Jun 16;23(6):1467. doi: 10.3390/molecules23061467 (PMC6099592; doi:10.3390/molecules23061467)
Supplement: Supplementary file 1 [file molecules-23-01467-s001.pdf]

Supplementary information

Identification and characterization of flavonoid biosynthetic enzyme genes in *Salvia miltiorrhiza*

Yuxing Deng [1], Caili Li [1], Heqing Li [1,2] and Shanfa Lu [1*]

[1] Institute of Medicinal Plant Development, Chinese Academy of Medical Sciences & Peking Union Medical College, No.151 Malianwa North Road, Haidian District, Beijing 100193, China; yuxingdeng2016@163.com (Y.D.); licaili390@163.com (C.L.); hqliaau@163.com (H.L.); sflu@implad.ac.cn (S.L.)

[2] College of Agronomy, Qingdao Agricultural University, No. 700 Changcheng Road, Chengyang District, Qingdao City, Shandong Province, 266109, China.

* Correspondence: sflu@implad.ac.cn (S.L.); Tel.: +86-10-57833366

Table S1. Sequence features of flavonoid metabolism pathway genes in *Arabidopsis thaliana* and other plants.

Gene name	Accession number	ORF (bp)	AA Len	MW (Da)	p*I*
AtCHS	AT5G13930	1188	395aa	43115.72	6.08
AtCHI	AT3G55120	741	246aa	26595.61	5.43
AtF3H	AT3G51240	1077	358aa	40275.72	5.29
AtFLS1	AT5G08640	1011	336aa	38281.77	5.63
AtFLS2	AT5G63580	753	250aa	28482.41	4.77
AtFLS3	AT5G63590	927	308aa	35451.53	5.13
AtFLS4	AT5G63595	840	279aa	31900.58	6.56
AtFLS5	AT5G63600	981	326aa	37092.85	4.66
AtFLS6	AT5G43935	882	293aa	33456.13	5.67
AtF3′H	AT5G07990	1542	513aa	56786.57	7.83
AtDFR	AT5G42800	1149	382aa	42774.96	5.43
AtANS	AT4G22880	1071	356aa	40396.42	5.23
CsF3′5′H	ABA40923.1	1533	510aa	57041.53	8.82
GmF3′5′H	AAM51564.1	1527	508aa	57185.75	9.11

Table S2. Primers used for full-length coding region cloning.

Gene name	Primer name	Sequence (5' to 3')
SmCHS1	CHS1-F	AACAAGAATGGTGACCGTGGA
	CHS1-R	TCAATTGATGTTGACGCTGTGCAG
SmCHS2	CHS2-F	CCAATGTGCAAGAATCCAATTTAGAG
	CHS2 -R	GTTGCACACCATGTTAAATATAGC
SmCHS3	CHS3 -F	ATGGCAAGCGTGGAGGAGATC
	CHS3 -R	TTATAAATCAATCGGCATGCTG
SmCHS4	CHS4 -F	ATGGCGACCGTGGAGGAGATC
	CHS4 -R	TTAATTAATCGGCATACTACGTAGC
SmCHS5	CHS5 -F	ATGGCAAGCGTGGAGGAGATC
	CHS5 -R	TTATAATTCAATCGGAATGCTGCGC
SmCHS6	CHS6 -F	TGTAATGTCGACAAAGCAGCAG
	CHS6 -R	GATAGTTAATGGAGGAGGCTTCGC
SmCHS7	CHS7-F	CAAACCCCGCCTAACAACAACG
	CHS7-R	CGTTATATTTAAATCGGCATGCTGTG
SmCHS8	CHS8-F	CAAACCCCGCCTAACAACAACG
	CHS8-R	GAACACAGACTAGAGGCCCGAGAC
SmCHI1	CHI1-F	CCACCTTCATCTTCATCTTCACC
	CHI1 -R	CATGTTTCTTGATTCATTGTGCCTC

Table S2. *Cont.*

SmCHI2	CHI2-F	CTTCATCTTCGCCTTCACCTTC
	CHI2-R	CATGTTTCTTGATTCATTGTGCCTC
SmCHI3	CHI3-F	GAGGCTGTGATGGTGGATGAAGTTC
	CHI3-R	TTATTTGGATAACTCGGCGGAGAGAG
SmCHI4	CHI4-F	CCATTGTTAACCACCTTCATCTTCAC
	CHI4 -R	CATGTTTCTTGATTCATTGTGCCTCA
SmF3H1	F3H1-F	CATGGCTGAAATGAAAATATCTCC
	F3H1-R	TGGTGGAATCAACCTTAATTTAGAA
SmF3H2	F3H2-F	ATGGCTGCACAAATGAAAATCTG
	F3H2-R	TAATAAAGGGTTGTTTTGGCTGGC
SmF3′5′H	F3′5′H-F	CAACATATAGTAGGCATGCAAGGTG
	F3′5′H-R	ACCTAGCTAGCTGCATAACAATGTG
SmF3′H1	F3′H1-F	ATATATTCACGTTGGCATGCA
	F3′H1-R	GCAAACACTTAAGCATGGTAAAC
SmF3′H2	F3′H 2-F	ACACACACATCACATATATGCAATG
	F3′H 2-R	TCAGATTTGGGCTTGATAAACATGG
SmF3′H3	F3′H 3-F	ATGCAGAGCTTCCACGAATTTCTAT
	F3′H 3-R	GCAACGGTACATTCATTCATCAAC
SmF3′H4	F3′H 4-F	CCTTGTCAATTAGTTTCGCTTCAG
	F3′H 4-R	CCAGTCATCACTCATACAAACTAGG
SmF3′H5	F3′H 5-F	CCTCTCAAACACAACTCTCATCG
	F3′H 5-R	CAAACCGAGGCATAAAATTCACATAG
SmF3′H6	F3′H 6-F	CTCTCTCCAAATTTAATTAGCAATGG
	F3′H 6-R	GCAGAGCCATAAATTCACATAG
SmFLS1	FLS1-F	CTATACCACCACCATGTAAGATCAC
	FLS1-R	CTCAAATCAGGGACAGTGGTTAT
SmFLS2	FLS2-F	AAATGGAGGTTGAGAGAGTGC
	FLS2-R	TATTTGTTTGGTGGGTAGAGTGA
SmFNS II	FLS II-F	GCGAGTTGTACTGATATGGAGCTAG
	FLS II-R	ACAGTAGCTAAACCGAATCAGGC
SmDFR	DFR-F	AATGCCGCTAGAAACCACCCCTC
	DFR-R	TCAATCACATTTGCCATTCTCGCCT
SmANS	ANS-F	CCATGCATTTCCACCCTAACATAC
	ANS-R	TACAACTCACACAATCAACTCGA

Table S3. Primers used for qRT-PCR.

Gene name	Primer name	Sequence (5' to 3')
SmUBQ10	UBQ10-RT-F	AGATGGGCGGACACTTGCTGATTA
	UBQ10-RT-R	ACTCTCCACCTCCAAAGTGATGGT
SmCHS1	CHS1-RT-F	CAAGCGCATGTGTGAAAAGTCGA
	CHS1-RT-R	TGCAGAAGACGAGGTGGGTGA
SmCHS2	CHS2-RT-F	ATGGGAATCGGTGAATTGTGTAA
	CHS2-RT-R	GCAAAGTCGGACAAGCTTCTG
SmCHS3	CHS3 –RT-F	GGCTTCTTATCGATCTCGTCAGA
	CHS3 –RT-R	CCAACCTTCGCCTCCACCTGAT
SmCHS4	CHS4 –RT-F	CATTTAAGCGCATGTGTGAGAGC
	CHS4 –RT-R	CATGTGGATGCTGCCGTTGGT
SmCHS5	CHS5 –RT-F	GTCTGCTGCGAGTCCACC
	CHS5 –RT-R	GATACCTTTTGCGAGATCTATGAGA
SmCHS6	CHS6 –RT-F	ATGTCGACAAAGCAGCAGCGC
	CHS6 –RT-R	TCGTCGTTTTGCAGAGGCGTTG
SmCHS7	CHS7-RT-F	CAAGCATCGCCGACTACGA
	CHS7-RT-R	CAAGTCGGCGGAAGTGGT
SmCHS8	CHS8-RT-F	CGTTTCCTGATTACTACTTCCGTGT
	CHS8-RT-R	CGGGATGTTGGGTTTTCTTTGA
SmCHI1	CHI1-RT-F	TGCCGATGAACTGGCCGATTCCA
	CHI1-RT-R	CCGTTAGTGGCAGAATCATAGTC
SmCHI2	CHI2-RT-F	AGTCTTCAAGGATGAAAGCTTCTC
	CHI2-RT-R	CCAAGCTCTGCCTCGTGGTA
SmCHI3	CHI3-RT-F	GCTCCTACCGTCCAGATTGTATT
	CHI3-RT-R	TTATTTGGATAACTCGGCGGA
SmCHI4	CHI4-RT-F	TTCCCACCCACAGCGAAGCCACG
	CHI4-RT-R	AATGAAGTCGGCGGAATCGG
SmF3H1	F3H1-RT-F	CAATGGGAGGTTCAAGAATGCAG
	F3H1-RT-R	GAAAAGAGGTTTGTTTTGGTTGGCG
SmF3H2	F3H2-RT-F	GACCCGGGATGACGGCAACACAT
	F3H2-RT-R	GAACCTCCCATTGCTTACATAAT
SmF3′5′H	F3′5′H-RT-F	ACTGAACTTGTTTACTGCAGGGAC
	F3′5′H-RT-R	GGTATGCTAGTTTCGGAATGTCG
SmF3′H1	F3′H1-RT-F	TTAGACAGGAGGAAGCGGGGATT
	F3′H1-RT-R	ACTCCTCCGCCTTCTCGTCTCCA

Table S3. *Cont.*

SmF3′H2	F3′H 2-RT-F	TCCGCCATGTTAGAGAGGAAGAAGT
	F3′H 2-RT-R	TCCACCATCGCCTTCAACTCTT
SmF3′H3	F3′H 3-RT-F	CCTTGGCTTAGTTTCCTCGATTTG
	F3′H 3-RT-R	TCAGTACCACCAGTTAGCAAGTCCT
SmF3′H4	F3′H 4-RT-F	CATGGATATGGTAGTAGGAGGAACG
	F3′H 4-RT-R	GCAGTTTGTTGATGTGGGATTCTT
SmF3′H5	F3′H 5-RT-F	AGACTGTAAGGTGGATGGGTATGA
	F3′H 5-RT-R	TCGAAATCTTGTCCCTTCACAT
SmF3′H6	F3′H 6-RT-F	ACATGACTGTAAGGTGGATGGGT
	F3′H 6-RT-R	TCGAAATCCTGTCCCTTCACAT
SmFLS1	FLS1-RT-F	CCTGATTATAGGGAGGTTACTGAAG
	FLS1-RT-R	ACCCCAAGAACCAAGTCCGG
SmFLS2	FLS2-RT-F	GGCGACCAAATTGAGATACTAAG
	FLS2-RT-R	TCACAAGCTTTGGAATTGGC
SmFNS II	FNSⅡ-RT-F	ATTCTGGATTTTTTTACCGCCG
	FNSⅡ-RT-R	CTCTCTGATCCCACCACTTTTGC
SmDFR	DFR-RT-F	GATGATCCCGAGAATGAAGTGATC
	DFR-RT-R	AGACACAAAGTACATCCATCCAGTC
SmANS	ANS-RT-F	CTGATTATATACCGGCTACATG
	ANS-RT-R	AATCCGCCGACTTCTTTCTC

Supplementary Table S4. The proteins used in the phylogenetic tree of CHS.

Protein name	Plant Species	Accession number
OsCHS1	*Oryza sativa*	A2ZEX7.1
OsCHS2	*Oryza sativa*	BAA19186.2
ZmCHS(C2)	*Zea mays*	CAA42764.1
ZmCHS(Whp)	*Zea mays*	CAA42763.1
ZmCHSL	*Zea mays*	NP_001149508.1
GhyCHS1	*Gerbera hybrida*	CAA86218.1
GhyCHS3	*Gerbera hybrida*	CAA86220.1
GhyCHS4	*Gerbera hybrida*	CAP20328.1
AtCHS	*Arabidopsis thaliana*	NP_196897.1
AtPKS-B	*Arabidopsis thaliana*	Q8LDM2.1
AtPKS-A	*Arabidopsis thaliana*	O23674.1
VvCHS1	*Vitis vinifera*	BAA31259.1
VvCHS2	*Vitis vinifera*	BAB84112.1
PcCHS	*Petroselinum crispum*	P16107.1
RgCHS1	*Ruta graveolens*	Q9FSB9.1
RgCHS2	*Ruta graveolens*	Q9FSB8.1
RgCHS3	*Ruta graveolens*	Q9FSB7.1
SvCHS	*Scutellaria viscidula*	ACC68839.1
SbaCHS1	*Scutellaria baicalensis*	AMW91735.1
SbaCHS2	*Scutellaria baicalensis*	AMW91736.1
AmCHS	*Antirrhinum majus*	CAA27338.1
ArCHS	*Agastache rugosa*	AFL72079.1
SsCHS	*Solenostemon scutellarioides*	ABP57071.1
PfCHS	*Perilla frutescens*	BAA19548.1
IpCHSA	*Ipomoea purpurea*	AAB02620.1
IpCHSB	*Ipomoea purpurea*	AAC49030.1
IpCHSC	*Ipomoea purpurea*	AAC49031.1
IpCHSD	*Ipomoea purpurea*	ABW69675.1
IpCHSE	*Ipomoea purpurea*	BAA87337.1
IpCHSFL1	*Ipomoea purpurea*	AAB41103.1
PhCHSA	*Petunia hybrida*	CAA32731.1
PhCHSB	*Petunia hybrida*	CAA32732.1
PhCHSD	*Petunia hybrida*	CAA32733.1
PhCHSF	*Petunia hybrida*	CAA32734.1
PhCHSG	*Petunia hybrida*	CAA32735.1
PhCHSH	*Petunia hybrida*	CAA32736.1
PhCHSJ	*Petunia hybrida*	CAA32737.1
SlCHS1	*Solanum lycopersicum*	CAA38980.1
SlCHS2	*Solanum lycopersicum*	CAA38981.1
MdCHS1	*Malus domestica*	AAY45748.1
MdCHS2	*Malus domestica*	AGE84303.1

Table S4. *Cont.*

MdCHS3	*Malus domestica*	ACJ54531
NnCHS	*Nelumbo nucifera*	ADD74168.1
GmCHS1	*Glycine max*	NP_001337038.1
GmCHS2	*Glycine max*	CAA46590.1
GmCHS3	*Glycine max*	NP_001276296.1
GmCHS4	*Glycine max*	CAA36317.1
GmCHS5	*Glycine max*	AAB01004.1
GmCHS6	*Glycine max*	AAA33951.1
GmCHS7	*Glycine max*	AAA33950.1
GmCHS8	*Glycine max*	NP_001304585.2
GmCHS9	*Glycine max*	ABQ63059.1
MsCHS1	*Medicago sativa*	AAA02823.1
MsCHS2	*Medicago sativa*	AAA02824.1
MsCHS3	*Medicago sativa*	CAA48226.1
MsCHS4	*Medicago sativa*	AAA02825.1
MsCHS8	*Medicago sativa*	AAA02826.1
MsCHS9	*Medicago sativa*	AAA02827.1
PnCHS	*Psilotum nudum*	BAA87922.1
EaCHS	*Equisetum arvense*	BAA89501.1
GbCHS	*Ginkgo biloba*	AAY52458.1
PstCHS	*Pinus strobus*	CAA06077.1
PsyCHS	*Pinus sylvestris*	CAA43166.1
PrCHS	*Pinus radiata*	AGY80771.1
PrCHSL	*Pinus radiata*	AAB80804.1
PhaCHS	*Phalaenopsis hybrida*	AAV70116.1
PpaCHS	*Physcomitrella patens*	ABB84527.1
NsCHSL	*Nicotiana sylvestris*	CAA74847.1

Table S5. The proteins used in the phylogenetic tree of CHI

Protein name	Plant Species	Accession number
AtCHI	*Arabidopsis thaliana*	At3g55120
AtCHIL	*Arabidopsis thaliana*	AT5g05270
AtFAP1	*Arabidopsis thaliana*	At3g63170
AtFAP2	*Arabidopsis thaliana*	At2g26310
AtFAP3	*Arabidopsis thaliana*	At1g53520
LjCHI1	*Lotus japonicus*	BAC53983.1
LjCHI2	*Lotus japonicus*	BAC53984.1
LjCHI3	*Lotus japonicus*	BAC54038.1
LjCHI4	*Lotus japonicus*	BAC53984.1
AhCHI I	*Arachis hypogaea*	AFP33452.1
AhCHI II	*Arachis hypogaea*	AJF45950.1
GmCHI1A	*Glycine max*	Glyma.20G241500.1
GmCHI1B1	*Glycine max*	Glyma.20G241600.1
GmCHI1B2	*Glycine max*	Glyma.10G292200.1
GmCHI2	*Glycine max*	Glyma.20G241700.1
GmCHI3A1	*Glycine max*	Glyma.13G262500.1
GmCHI3A2	*Glycine max*	Glyma.15G242900.1
GmCHI3B1	*Glycine max*	Glyma.03G154600.1
GmCHI3B2	*Glycine max*	Glyma.19G156900.1
GmCHI3C1	*Glycine max*	Glyma.14G098100.1
GmCHI3C2	*Glycine max*	Glyma.17G226600.1
GmCHI4A	*Glycine max*	Glyma.06G143000.1
GmCHI4B	*Glycine max*	Glyma.04G222400.1
VvCHI1	*Vitis vinifera*	P51117.1
VvCHI2	*Vitis vinifera*	A5ANT9.1
PfCHI	*Perilla frutescens*	BAG14301.1
SbaCHI	*Scutellaria baicalensis*	ADQ13184.1
ArCHI	*Agastache rugosa*	AFL72080.1
InCHIL	*Ipomoea nil*	BAO58578.1
GhCHI	*Gossypium hirsutum*	ABM64798.1
SmeCHI	*Saussurea medusa*	AAM48130.1
CsCHI	*Camellia sinensis*	AAZ17563.2
DcCHI	*Dianthus caryophyllus*	CAA91931.1
PcCHI	*Pyrus communis*	ABQ08639.1
IpCHI	*Ipomoea purpurea*	ABW69677.1
PhCHIA	*Petunia hybrida*	AAF60296.1
PhCHIB	*Petunia hybrida*	P11651.1
RsCHI	*Raphanus sativus*	O22651.1
NtCHI	*Nicotiana tabacum*	Q33DL3.1
ZmCHI	*Zea mays*	Q08704.1
HvCHI	*Hordeum vulgare*	AAM13449.1

Table S5. *Cont.*

OsCHI	*Oryza sativa*	AAM13448.1
SlCHI1	*Solanum lycopersicum*	Solyc05g010320.2.1
SlCHI2	*Solanum lycopersicum*	Solyc05g010310.2.1
SlCHI3	*Solanum lycopersicum*	Solyc05g052240.2.1
SlCHI4	*Solanum lycopersicum*	Solyc07g062030.2.1
SlCHI5	*Solanum lycopersicum*	Solyc08g061480.2.1
SlCHI6	*Solanum lycopersicum*	Solyc02g067870.2.1
SlCHI7	*Solanum lycopersicum*	Solyc06g084260.2.1
MsCHI	*Medicago sativa*	P28012.1
PvCHI	*Phaseolus vulgaris*	P14298.2
PlCHI	*Pueraria lobata*	Q43056.1
LaCHIL1	*Lupinus angustifolius*	CCM80407.1
LaCHIL2	*Lupinus angustifolius*	CCM80406.1

Table S6. The proteins used in the phylogenetic tree of FNSII, F3′5′H and F3′H

Protein name	Plant Species	Accession number
PfFNSII	*Perilla frutescens*	BAB59004.1
SbaFNSII-1	*Scutellaria baicalensis*	AMW91728.1
SbaFNSII-2	*Scutellaria baicalensis*	AMW91729.1
ThFNSII	*Torenia hybrida*	BAA84072
AmFNSII	*Antirrhinum majus*	BAA84071.1
GtFNSII	*Gentiana triflora*	BAD91809.1
GmFNSII	*Glycine max*	ACV65037.1
LeFNSII	*Lobelia erinus*	BAF49323.1
CcFNSII	*Callistephus chinensis*	AAF04115.1
PoFNSII	*Pilosella officinarum*	ACB56919.1
GhyFNSII	*Gerbera hybrida*	AAD39549.1
CsFNSII	*Camellia sinensis*	ACH99109.1
GeFNSII	*Glycyrrhiza echinata*	P93149.2
MtFNSII	*Medicago truncatula*	ABC86159.1
SbFNSII	*Sorghum bicolor*	XP_002461286.1
OsFNSII	*Oryza sativa*	BAG94859.1
ZmFNSII	*Zea mays*	XP_008663013.1
GmF3'H	*Glycine max*	BAD97828.1
MtF3'H	Medicago truncatula	XP_003598945.1
MdF3'H	*Malus domestica*	ACR14867.1
GhF3'H	*Gossypium hirsutum*	ADP24159.1
BnF3'H	*Brassica napus*	ABC58722.1
AtF3'H	*Arabidopsis thaliana*	Q9SD85.1
ThF3'H	*Torenia hybrida*	BAB87838.1
AmF3'H	Antirrhinum majus	ABB53383.1
PfF3'H	*Perilla frutescens*	BAB59005.1
GtF3'H	*Gentiana triflora*	BAD91808
IbF3'H	*Ipomoea batatas*	AEH42499.1
InF3'H	*Ipomoea nil*	BAD00190
IpF3'H	*Ipomoea purpurea*	AAR00229.1
SlF3'H	Solanum lycopersicum	NP_001289844.1
PhF3'H (Ht1)	*Petunia hybrida*	Q9SBQ9.1
LeF3'H	*Lobelia erinus*	BAF49324.1
VvF3'H1	*Vitis vinifera*	BAE47003.1
VvF3'H2	*Vitis vinifera*	BAE47003.2
VvF3'H3	*Vitis vinifera*	BAE47003.3
VvF3'H4	*Vitis vinifera*	BAE47003.4
CsF3'H1	*Camellia sinensis*	AKJ86992
CsF3'H2	*Camellia sinensis*	AKM12329.1
CsF3'H3	*Camellia sinensis*	AKM12330.1

Table S6. *Cont.*

CnF3'H	*Camellia nitidissima*	ADZ28515.1
OhF3'H	*Osteospermum hybrida*	ABB29899.1
GhyF3'H	*Gerbera hybrida*	ABA64468
EsF3'H	*Epimedium sagittatum*	ADE80941.1
SbF3'H1	*Sorghum bicolor*	ABG54319.1
SbF3'H2	*Sorghum bicolor*	ABG54320.1
SbF3'H3	*Sorghum bicolor*	ABG54321.1
ZmF3'H	*Zea mays*	AEF33624.1
GbF3'H-like	*Ginkgo biloba*	AJO67233.1
SiF3'H-like	*Sesamum indicum*	XP_011095827.1
PhyF3'5'H	*Phalaenopsis hybrida*	AAZ79451.1
DgF3'5'H	*Delphinium grandiflorum*	AAX51796.1
LeF3'5'H	*Lobelia erinus*	BAF49321.1
CmF3'5'H	*Campanula medium*	O04773
EsF3'5'H	*Epimedium sagittatum*	ADE80942.1
GhiF3'5'H	*Gossypium hirsutum*	AAP31058
VvF3'5'H	*Vitis vinifera*	ABH06585
CpF3'5'H	*Cyclamen persicum*	ACX37698.1
CsF3'5'H	*Camellia sinensis*	ABA40923.1
CtF3'5'H	*Clitoria ternatea*	BAE72870
GmF3'5'H	*Glycine max*	AAM51564.1
VwF3'5'H	*Viola wittrockiana*	BAF93855.1
EeF3'5'H	*Eustoma exaltatum*	Q96418
GtF3'5'H	*Gentiana triflora*	BAA12735
VmF3'5'H	*Vinca major*	BAC97831.1
CrF3'5'H	*Catharanthus roseus*	CAA09850
PhF3'5'H (Hf1)	*Petunia hybrida*	CAA80266.1
PhF3'5'H1 (Hf2)	*Petunia hybrida*	CAA80265.1
StF3'5'H	*Solanum tuberosum*	AAV85470.1
SlF3'5'H	*Solanum lycopersicum*	ADC80513.1
SmeF3'5'H	*Solanum melongena*	P37120
ThF3'5'H	*Torenia hybrida*	BAB20076
AkF3'5'H	*Antirrhinum kelloggii*	BAJ16329.1
GlhF3'5'H	*Glandularia hybrida*	AAT34974.1

Table S7. The proteins used in the phylogenetic tree of F3H, FLS and ANS

Protein name	Plant Species	Accession number
GbF3H	*Ginkgo biloba*	AAU93347.1
ZmANS	*Zea mays*	NP_001106074.1
SbANS	*Sorghum bicolor*	XP_002451336.1
OsANS	*Oryza sativa*	CAA69252.1
TaANS	*Triticum aestivum*	BAE98276.1
AcANS1	*Allium cepa*	ABR24157.1
AcANS2	*Allium cepa*	ABM66367.1
SsANS	*Solenostemon scutellarioides*	ABP57079.1
PfANS	*Perilla frutescens*	BAA20143.1
AtANS	*Arabidopsis thaliana*	NP_194019.1
NtANS1	*Nicotiana tabacum*	AFM52334.1
NtANS2	*Nicotiana tabacum*	AFM52335.1
PhANS	*Petunia hybrida*	P51092.1
StANS1	*Solanum tuberosum*	NP_001274859.1
StANS2	*Solanum tuberosum*	AEJ90548.1
MtANS	*Medicago truncatula*	ABU40983.1
GmANS	*Glycine max*	AAR26525.1
EgANS	*Eustoma grandiflorum*	BAJ08929.2
TcANS	*Theobroma cacao*	ADD51355.1
VvANS	*Vitis vinifera*	NP_001268147.1
CsANS	*Citrus sinensis*	NP_001275784.1
FaANS	*Fragaria ananassa*	AAU12369.1
PpANS	*Prunus persica*	ABX89943.1
MdANS	*Malus domestica*	AAD26205.1
PcANS	*Pyrus communis*	ABB70119.1
GmF3H1	*Glycine max*	NP_001236797.1
GmF3H2	*Glycine max*	AAU06218.1
VvF3H	*Vitis vinifera*	CAA53579.1
AtF3H	*Arabidopsis thaliana*	NP_190692.1
MtF3H	*Medicago truncatula*	ACR15123.1
StF3H	*Solanum tuberosum*	NP_001274930.1
PhF3H	*Petunia hybrida*	AAC49929.1
CsF3H	*Citrus sinensis*	BAA36553.1
FaF3H	*Fragaria ananassa*	BAE17126.1
PpF3H	*Prunus persica*	AQX36284.1
PcF3H	*Pyrus communis*	AGL81347.1
MdF3H	*Malus domestica*	BAB92997.1
EgF3H	*Eustoma grandiflorum*	BAD34459
PcrF3H	*Petroselinum crispum*	AAP57394.1
AcF3H	*Allium cepa*	AAO63022.1
NtF3H	*Nicotiana tabacum*	AAC15414.1

Table S7. *Cont.*

AmF3H	*Antirrhinum majus*	BAX37181.1
SsF3H	*Solenostemon scutellarioides*	ABP57073.1
PfF3H	*Perilla frutescens*	BAA19657.1
OsF3H	*Oryza sativa*	NP_001054157.1
TaF3H	*Triticum aestivum*	ABR13013.1
SbF3H	*Sorghum bicolor*	ADB66755.1
ZmF3H1	*Zea mays*	NP_001130275.1
ZmF3H2	*Zea mays*	AAA91227.1
TcF3H	*Theobroma cacao*	EOX90852.1
GbF3H	*Ginkgo biloba*	AAU93347.1
MdFLS	*Malus domestica*	AAX89401.1
PpFLS	*Prunus persica*	AJO70134.1
PcFLS	*Pyrus communis*	ABB70118.1
FaFLS	*Fragaria ananassa*	ABH07784.1
MtFLS1	*Medicago truncatula*	AES97739.1
MtFLS2	*Medicago truncatula*	AES71332.1
GmFLS	*Glycine max*	NP_001237419.1
VvFLS	*Vitis vinifera*	BAE75807.1
AmFLS	*Antirrhinum majus*	ABB53382.1
EgFLS	*Eustoma grandiflorum*	BAD34463.1
PcrFLS	*Petroselinum crispum*	AAP57395.1
StFLS	*Solanum tuberosum*	CAA63092.1
PhFLS	*Petunia hybrida*	CAA80264.1
NtFLS	*Nicotiana tabacum*	ABE28017.1
AcFLS	*Allium cepa*	AQR58516.1
TcFLS	*Theobroma cacao*	EOY09743.1
CsFLS1	*Citrus sinensis*	XP_006485472.1
CsFLS2	*Citrus sinensis*	XP_006466183.1
AtFLS1	*Arabidopsis thaliana*	NP_196481.1
AtFLS2	*Arabidopsis thaliana*	NP_201163.1
AtFLS3	*Arabidopsis thaliana*	NP_201164.1
AtFLS4	*Arabidopsis thaliana*	NP_680463.1
AtFLS5	*Arabidopsis thaliana*	NP_001032131.1
AtFLS6	*Arabidopsis thaliana*	NP_680388.1
OsFLS	*Oryza sativa*	BAD17324.1
TaFLS	*Triticum aestivum*	AHW49495.1
ZmFLS	*Zea mays*	NP_001140915.1
SbFLS	*Sorghum bicolor*	EES07584.1
GbFLS	*Ginkgo biloba*	ACY00393.1

Table S8. The proteins used in the phylogenetic tree of DFR

Protein name	Plant Species	Accession number
FhDFR1	*Freesia hybrida*	APG32498.1
FhDFR2	*Freesia hybrida*	APG32494.1
FhDFR3	*Freesia hybrida*	APG32495.1
IhDFR	*Iris hollandica*	BAF93856.1
ApDFR	*Agapanthus praecox*	BAE78769.1
BfDFR	*Bromheadia finlaysoniana*	AAB62873.1
LhDFR	*Lilium hybrida*	BAB40789.1
ZmDFR	*Zea mays*	NP_001152467.2
OsDFR	*Oryza sativa*	BAA36182.1
HvDFR	*Hordeum vulgare*	P51106.1
TaDFR1	*Triticum aestivum*	AAO60213.1
TaDFR2	*Triticum aestivum*	AAQ77347.1
AtrDFR	*Amborella trichopoda*	XP_006878526.1
GbDFR	*Ginkgo biloba*	AGR34043.1
LjDFR1	*Lotus japonicus*	BAE19948.1
LjDFR2	*Lotus japonicus*	BAE19949.1
LjDFR3	*Lotus japonicus*	BAE19950.1
LjDFR4a	*Lotus japonicus*	BAE19951.1
LjDFR4b	*Lotus japonicus*	BAE19952.1
LjDFR5	*Lotus japonicus*	BAE19953.1
MtDFR1	*Medicago truncatula*	AAR27014.1
MtDFR2	*Medicago truncatula*	AAR27015.1
GmDFR1	*Glycine max*	NP_001238612.2
GmDFR2	*Glycine max*	ABM64803.1
RhDFR	*Rosa hybrida*	BAA12723.1
FaDFR	*Fragaria ananassa*	AAC25960.1
MdDFR	*Malus domestica*	AAO39816.1
VvDFR	*Vitis vinifera*	NP_001268144.1
AtDFR	*Arabidopsis thaliana*	BAA85261.1
DcDFR	*Dianthus caryophyllus*	CAA91924.1
GhDFR1	*Gossypium hirsutum*	AHG97389.1
GhDFR2	*Gossypium hirsutum*	ACV72642.1
PtDFR1	*Populus trichocarpa*	XP_002300759.1
PtDFR2	*Populus trichocarpa*	XP_002307667.2
PhDFR	*Petunia hybrida*	AAF60298.1
NtDFR	*Nicotiana tabacum*	ABN80437.1
SlDFR	*Solanum lycopersicum*	NP_001234408.1
InDFR	*Ipomoea nil*	BAA22072.1
IpDFR	*Ipomoea purpurea*	BAA74700.1
CsDFR	*Camellia sinensis*	BAA84940.1
VmDFR	*Vaccinium macrocarpon*	AAL89714.1

Table S8. *Cont.*

GjDFR	*Gerbera jamesonii*	AHF58605.1
GhyDFR	*Gerbera hybrid cultivar*	CAA78930.1
AmDFR	*Antirrhinum majus*	CAA33543.1
AaDFR	*Angelonia angustifolia*	AHM27144.1
PbDFR	*Penstemon barbatus*	AIY51701.1
PfDFR	*Perilla frutescens*	BAA19658.1
SsDFR	*Solenostemon scutellarioides*	ABP57077.1

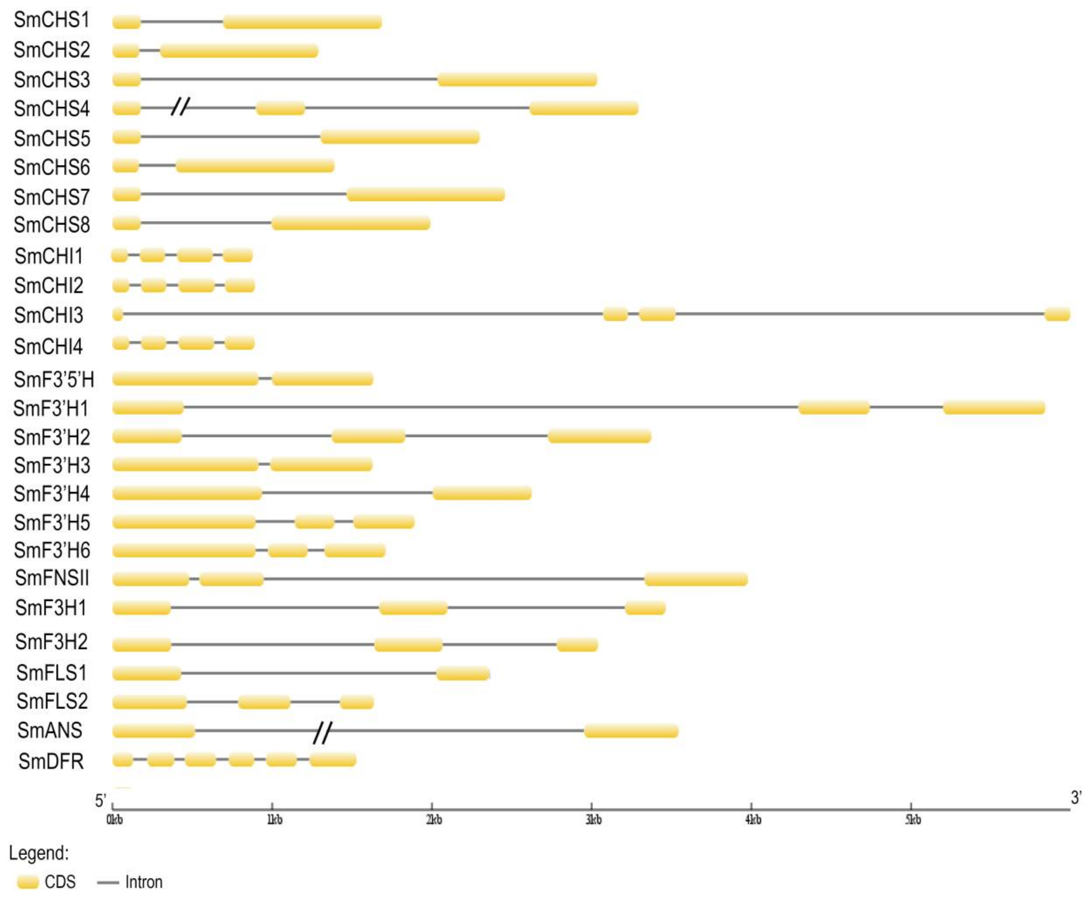

Figure S1. Exon/intron Structures of flavonoid biosynthesis related genes. Thick yellow lines represent exons. Thin gray lines represent introns. Unknown sequences in introns of *SmCHS4* and *SmANS* are indicated by '//'.

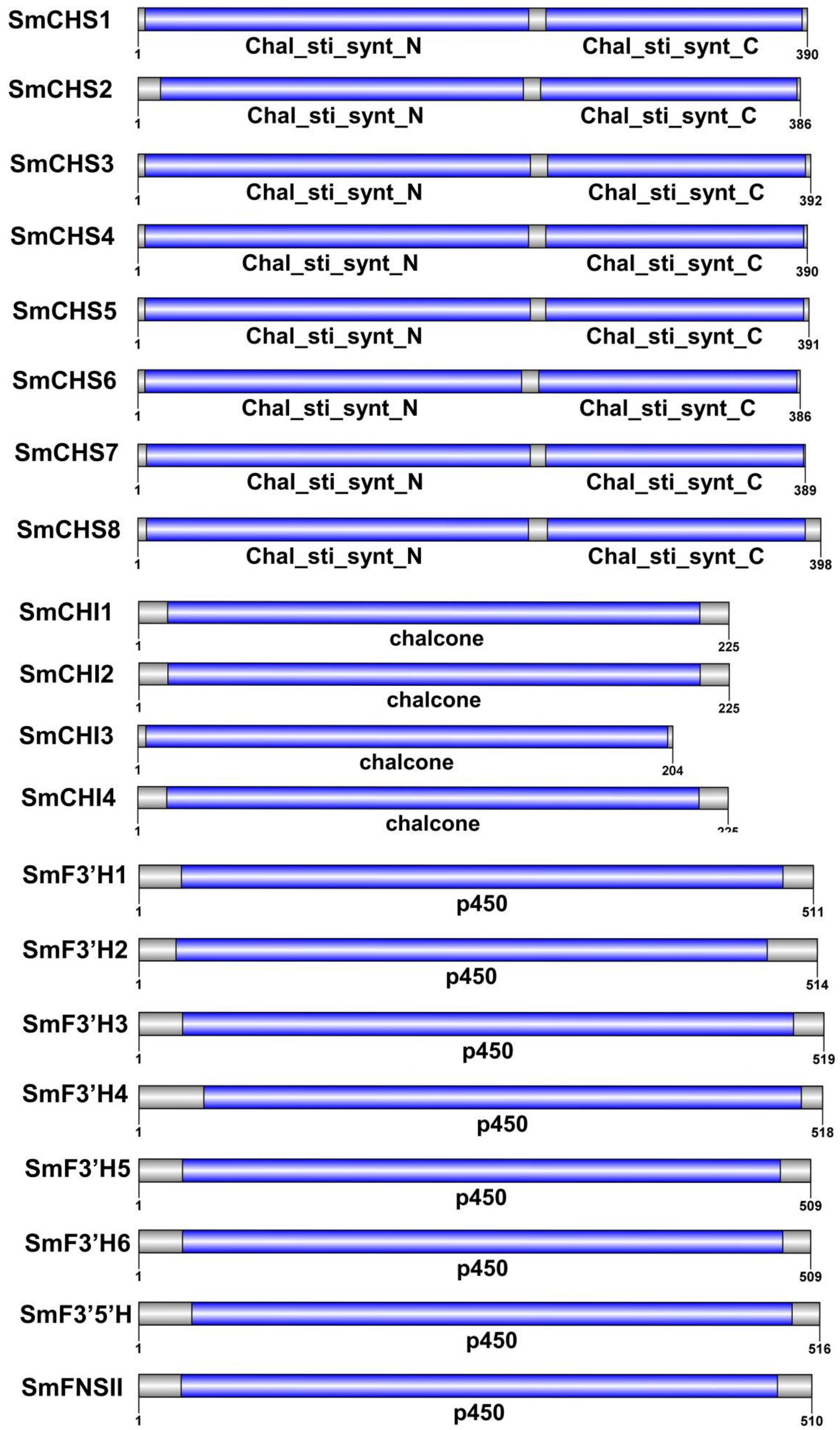
SmCHS1
Chal_sti_synt_N
Chal_sti_synt_C
1
390
SmCHS2
Chal_sti_synt_N
Chal_sti_synt_C
1
386
SmCHS3
Chal_sti_synt_N
Chal_sti_synt_C
1
392
SmCHS4
Chal_sti_synt_N
Chal_sti_synt_C
1
390
SmCHS5
Chal_sti_synt_N
Chal_sti_synt_C
1
391
SmCHS6
Chal_sti_synt_N
Chal_sti_synt_C
1
386
SmCHS7
Chal_sti_synt_N
Chal_sti_synt_C
1
389
SmCHS8
Chal_sti_synt_N
Chal_sti_synt_C
1
398
SmCHI1
chalcone
1
225
SmCHI2
chalcone
1
225
SmCHI3
chalcone
1
204
SmCHI4
chalcone
1
SmF3'H1
p450
1
511
SmF3'H2
p450
1
514
SmF3'H3
p450
1
519
SmF3'H4
p450
1
518
SmF3'H5
p450
1
509
SmF3'H6
p450
1
509
SmF3'5'H
p450
1
516
SmFNSII
p450
1
510

Figure S2. *Cont*.

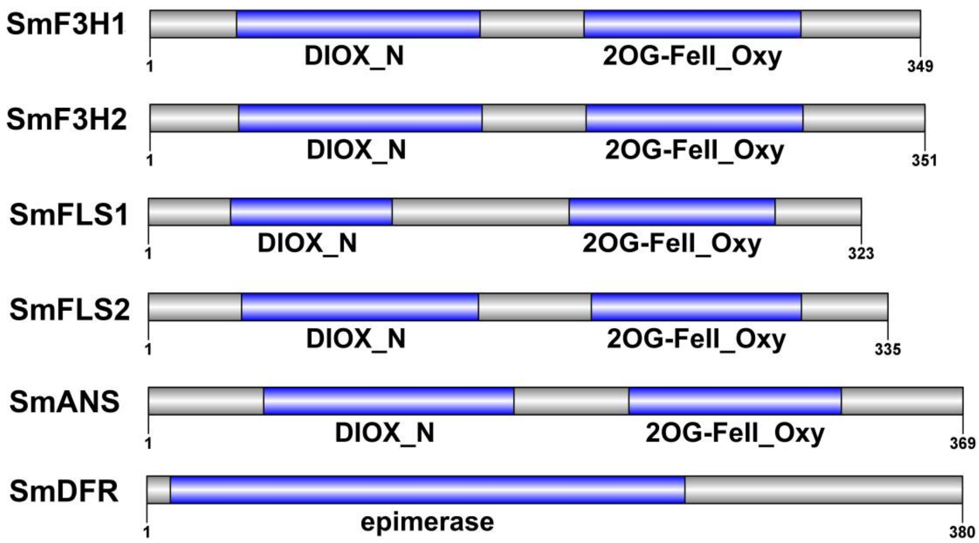

Figure S2. Conserved domains in flavonoid biosynthesis related proteins in *S. miltiorrhiza*. Conserved domains were predicated by searching Pfam and shown in blue boxes. Names of conserved domains are indicated below the boxes.

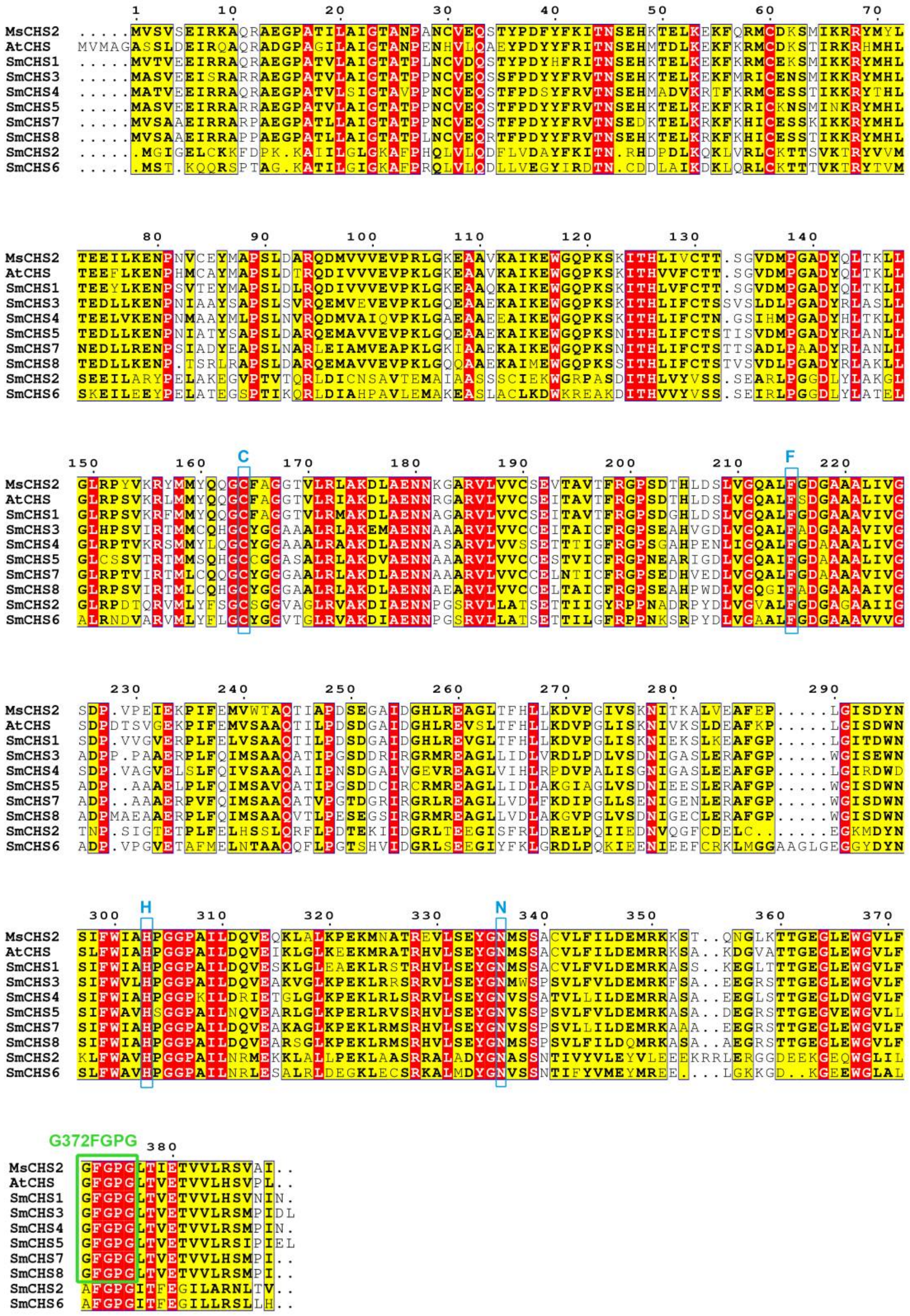

Figure S3. Amino acid sequence alignment of SmCHS1 –SmCHS8 against *Medicago sativa* MsCHS2, and *Arabidopsis thaliana* AtCHS. Identical sites were shown in white letters on a red background, conserved sites were shown in black letters on a yellow background and others were shown in white background. The catalytic triad C164-H303-N336 and the gatekeeper F215 were boxed and marked in blue letters. The highly conserved CHS signature sequence, G372FGPG were boxed and marked in green letters. The figure was produced using the ESPript server (http://espript.ibcp.fr/ESPript/ESPript/).

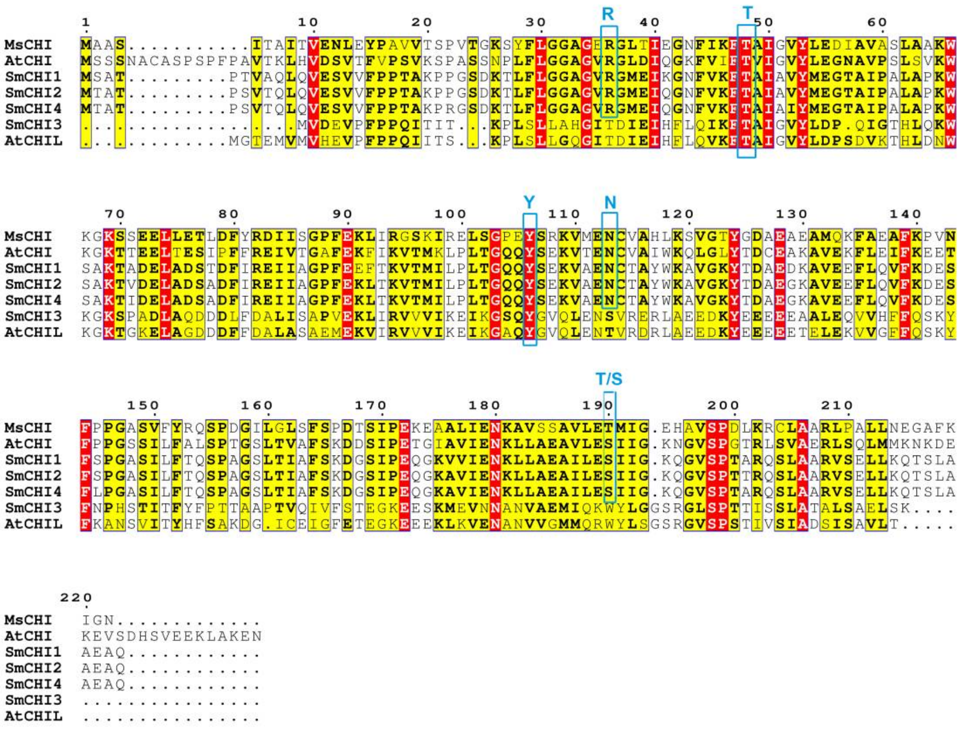

Figure S4. Amino acid sequence alignment of SmCHI1–SmCHI4 against *Medicago sativa* MsCHI, *Arabidopsis thaliana* AtCHI and AtCHIL. Residue numbering refers to the MsCHI sequence. Identical sites were shown in white letters on a red background, conserved sites are shown in black letters on a yellow background and others are shown in white background. Critical catalytic residue of *bona fide* CHI are boxed and indicated in blue letters. Among them, T190 are legume specific whereas S190 are much more conserved in other non-legume plant species. The figure was produced using the ESPript server (http://espript.ibcp.fr/ESPript/ESPript/)

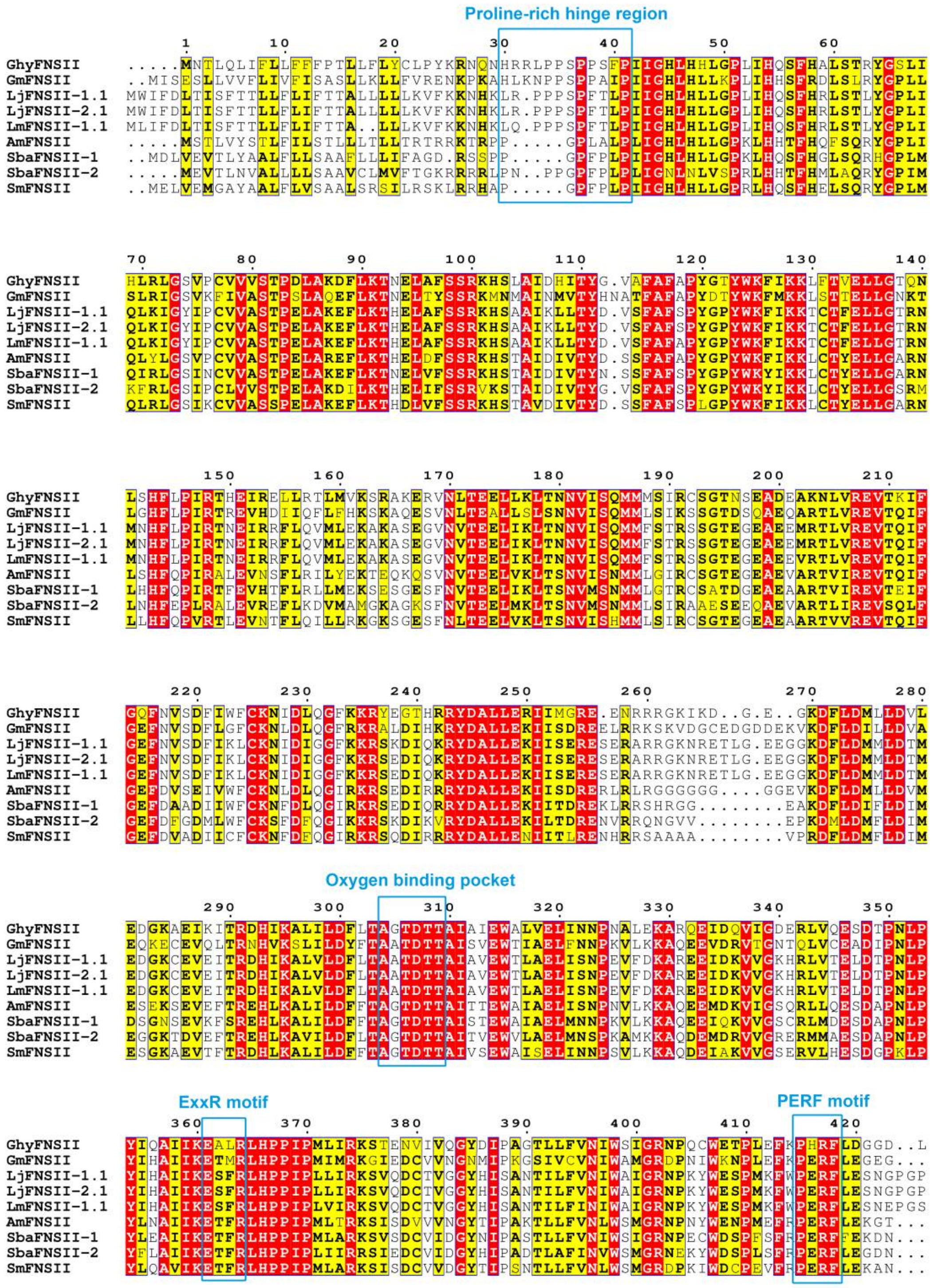
Proline-rich hinge region
Oxygen binding pocket
ExxR motif
PERF motif
GhyFNSII
GmFNSII
LjFNSII-1.1
LjFNSII-2.1
LmFNSII-1.1
AmFNSII
SbaFNSII-1
SbaFNSII-2
SmFNSII

Figure S5. *Cont*.

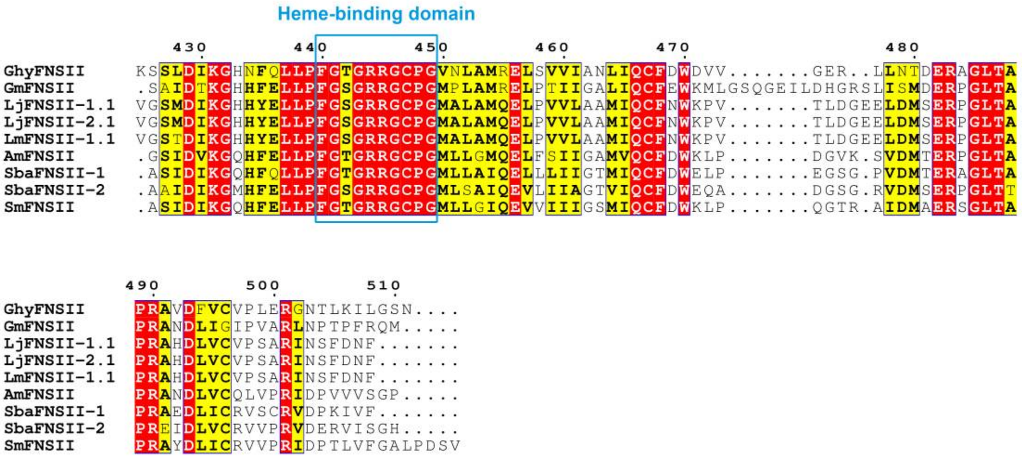

Figure S5. Comparison of SmFNSII proteins with other plant FNSII proteins. The amino acid sequences of SmFNSII was aligned with the amino acid sequences from *Gerbera hybrid* GhyFNSII, *Glycine max* GmFNSII, *Lonicera japonica* LjFNSII-1.1 and LjFNSII-2.1, *Lonicera macranthoides* LmFNSII-1.1, *Antirrhinum majus* AmFNSII and *Scutellaria baicalensis* SbaFNSII-1, SbaFNSII-2. Identical sites were shown in white letters on a red background, conserved sites are shown in black letters on a yellow background and others are shown in white background. P450s have conserved motifs including proline rich hinge region, oxygen binding pocket, E-R-R triade consisting of the E and R from the ExxR in the K-helix and the R from the "PERF" consensus sequence, and heme-binding pocket are boxed in blue. They are universal motifs among P450s. Among them, the E-R-R triad and the cysteine in the heme-binding domain are highly conserved in all primary amino acid sequence of plant P450s. The figure was produced using the ESPript server (http://espript.ibcp.fr/ESPript/ESPript/).

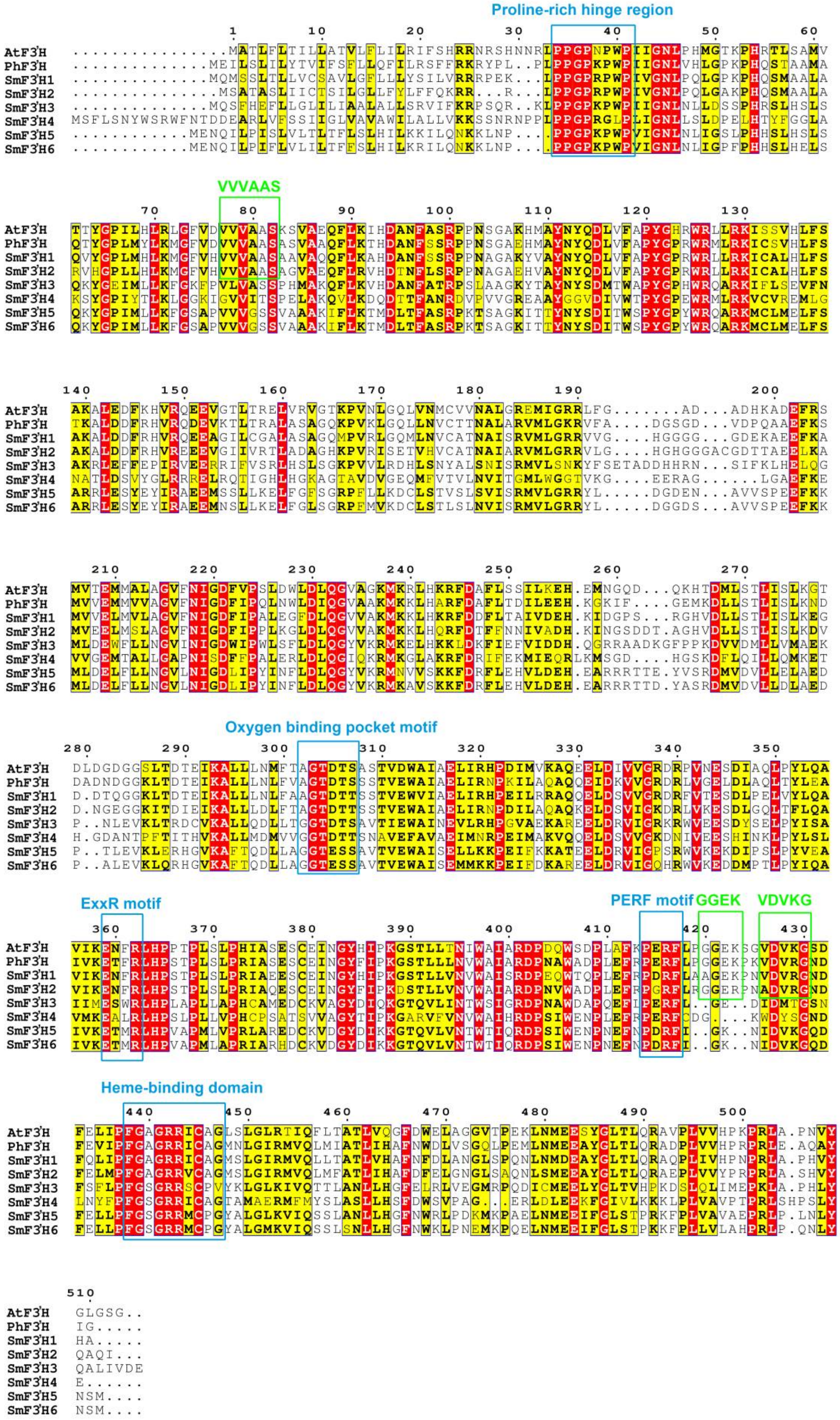
Proline-rich hinge region
VVVAAS
Oxygen binding pocket motif
ExxR motif
PERF motif
GGEK
VDVKG
Heme-binding domain
AtF3'H
PhF3'H
SmF3'H1
SmF3'H2
SmF3'H3
SmF3'H4
SmF3'H5
SmF3'H6

Figure S6. Amino acid sequence alignment of SmF3′H1–SmF3′H6 against *Arabidopsis thaliana* AtF3′H from and Petunia hybrid PhF3′H. Identical sites were shown in white letters on a red background, conserved sites are shown in black letters on a yellow background and others are shown in white background. The P450-featured conserved motif, including the proline-rich "hinge" region (P/I)PGPx(G/P)xP, oxygen binding pocket motif, E-R-R triade consisting of the EXXR and PERF motif and heme binding domain are boxed and indicated in blue letters. Three F3′H-specific conserved motifs "VVVAAS", "GGEK" and "VDVKG" have similarity counterparts at the corresponding sites of SmF3′H1 and SmF3′H2. They are boxed and indicated in green. The figure was produced using the ESPript server (http://espript.ibcp.fr/ESPript/ESPript/).

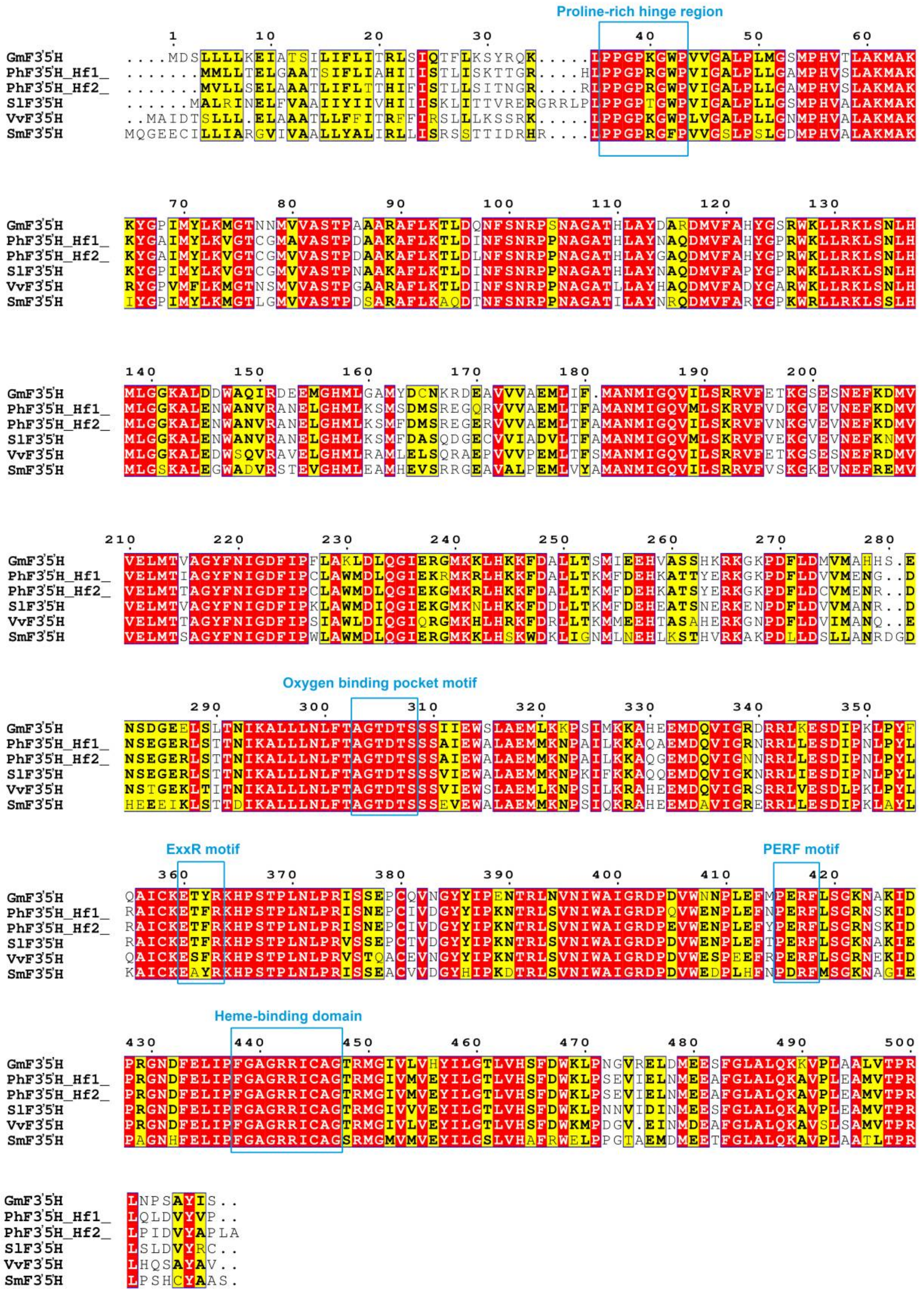

Figure S7. Amino acid sequence alignment of SmF3′5′H against *Glycine max* GmF3′5′H, *Petunia hybrid* PhF3'5'H (Hf1) and PhF3'5'H (Hf2), *Solanum lycopersicum* SlF3′5′H, *Vitis vinifera* VvF3′5′H. The P450-featured conserved motif, including the proline-rich "hinge" region, oxygen binding pocket motif, E-R-R triade consisting of the EXXR and PERF motif and heme binding domain are boxed and indicated in blue letters. The figure was produced using the ESPript server (http://espript.ibcp.fr/ESPript/ESPript/).

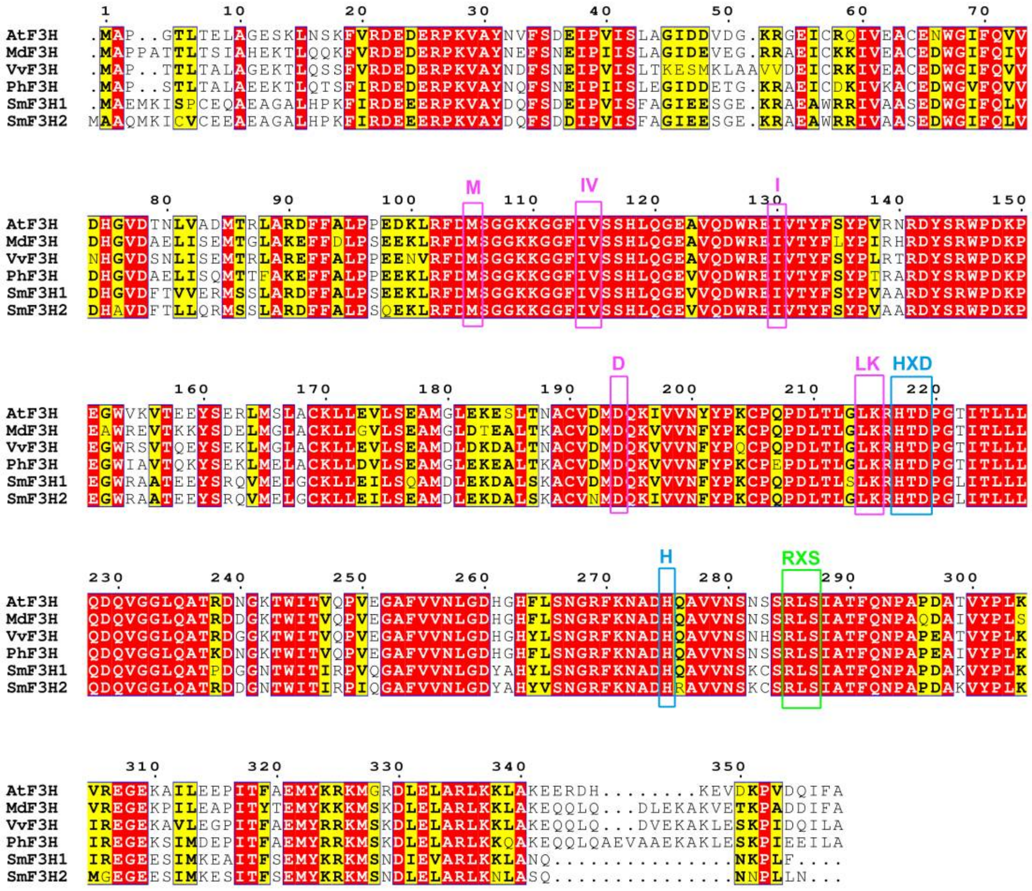

Figure S8. Amino acid sequence alignment of SmF3H1 and SmF3H2 against other plant F3Hs. The amino acid sequences of F3H from *S. miltiorrhiza* was aligned with the amino acid sequences from *Malus domestica* MdF3H, *Vitis vinifera* F3H, and *Petunia hybrid* PhF3H. The amino acid residues involved in ferrus iron binding and 2-oxoglutarate binding are indicated in blue and green box. Seven highly conserved residues with critical roles in determing the activity of F3H are indicated in pink box. The figure was produced using the ESPript server (http://espript.ibcp.fr/ESPript/ESPript/).

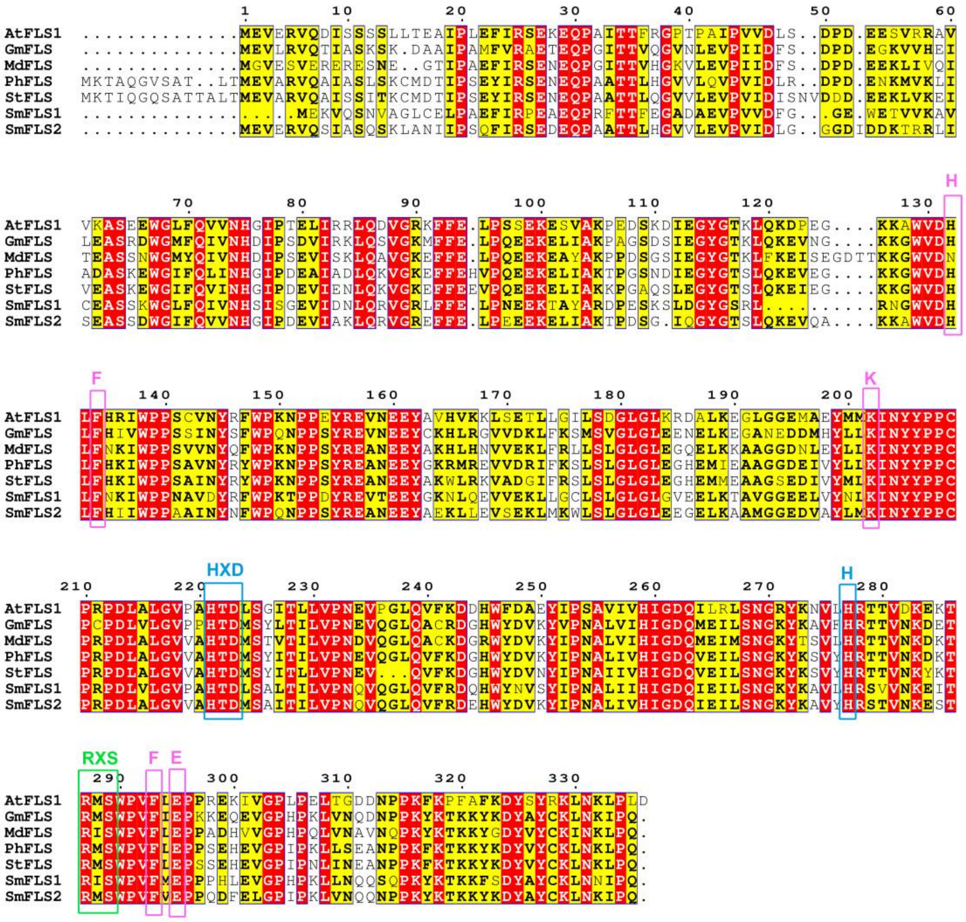

Figure S9. Amino acid sequence alignment of SmFLS1 and SmFLS2 against other plant FLSs. The amino acid sequences of FLS from *S. miltiorrhiza* was aligned with the amino acid sequences from *Malus domestica* MdFLS, *Vitis vinifera* FLS, *Petunia hybrid* PhFLS and *Solanum tuberosum* StFLS. The amino acid residues involved in ferrus iron binding and 2-oxoglutarate binding are indicated in blue and green box, respectively. Five potential substrate binding residues are indicated in pink box. The figure was produced using the ESPript server (http://espript.ibcp.fr/ESPript/ESPript/).

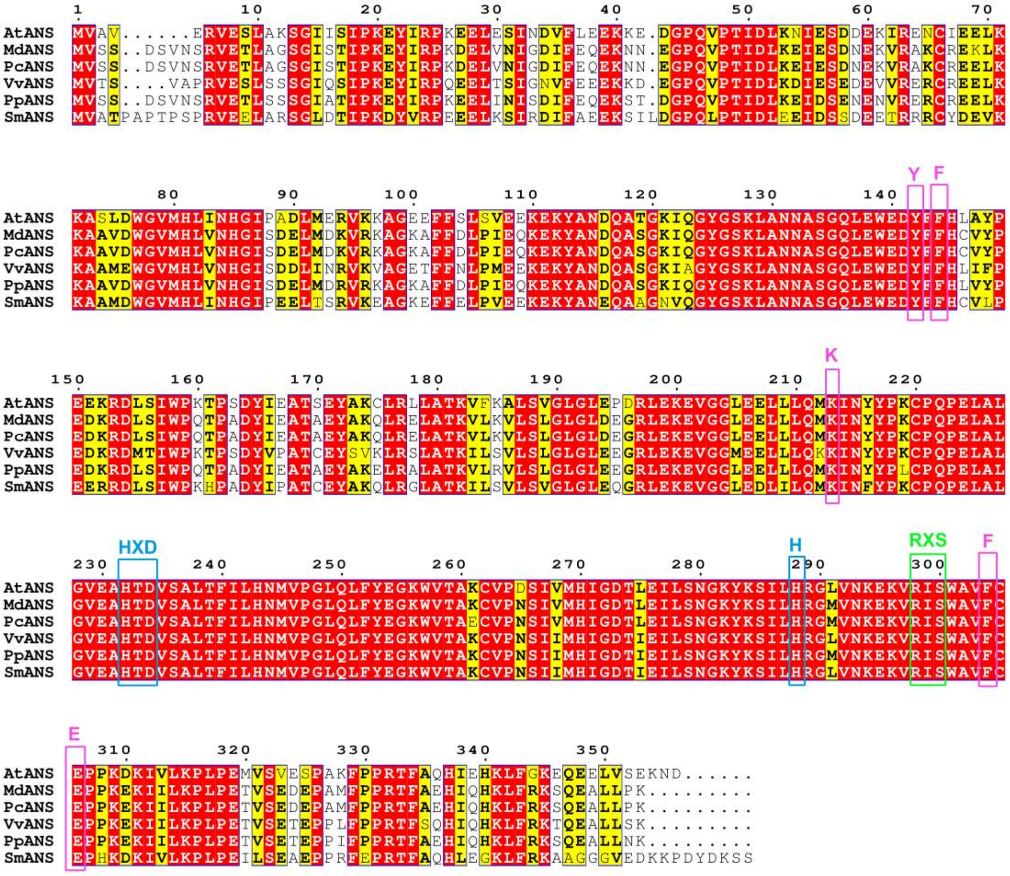

Figure S10. Amino acid sequence alignment of SmANS against other plant ANSs. The amino acid sequences of ANS from *S. miltiorrhiza* was aligned with the amino acid sequences from *Malus domestica* MdANS, Pyrus communis PcANS, *Vitis vinifera* VvANS and *Prunus persica* PpANS. The amino acid residues involved in ferrus iron binding and 2-oxoglutarate binding are indicated in blue and green box, respectively. Five potential substrate binding residues are indicated in pink box. The figure was produced using the ESPript server (http://espript.ibcp.fr/ESPript/ESPript/).

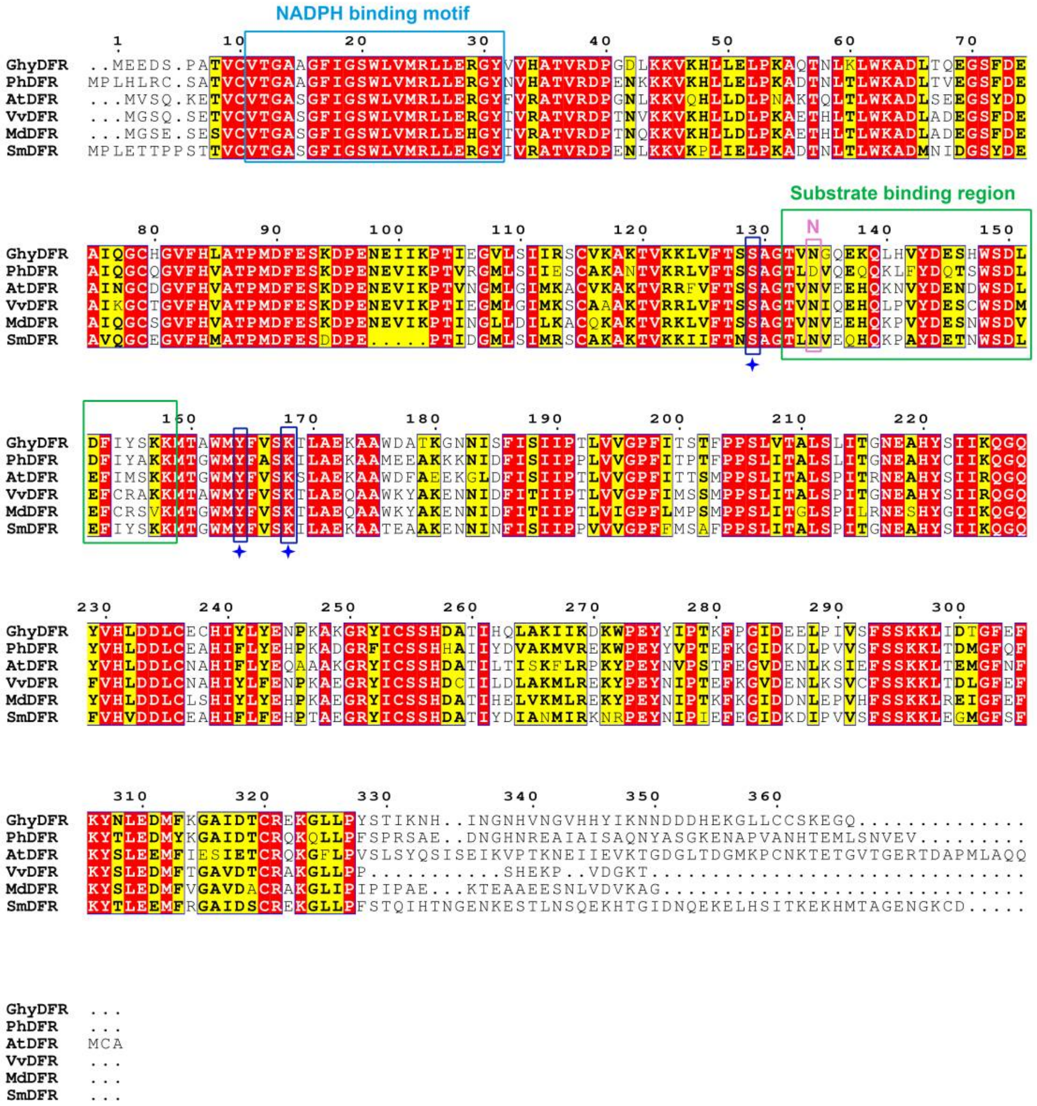

Figure S11. Amino acid sequence alignment of SmDFR against other plant DFRs. The amino acid sequences of DFR from *S. miltiorrhiza* was aligned with the amino acid sequences from *Gerbera hybrid* GhyDFR, *Petunia hybrida* PhDFR, *Arabidopsis thaliana* AtDFR, *Vitis vinifera* VvDFR and *Malus domestica* DFR. The conserved catalytic triad site, S129, Y164 and K168 are boxed in dark blue and indicated in blue stars. NADPH-binding motif is boxed in light blue. The substrate binding region is indicated in green box. The amino acid residue at the 134th position is particularly important for the substrate specificity. It has been indicated in pink box. The figure was produced using the ESPript server (http://espript.ibcp.fr/ESPript/ESPript/).
